# Temporal dynamics predict symptom onset and cognitive decline in familial frontotemporal dementia

**DOI:** 10.1002/alz.12824

**Published:** 2022-11-15

**Authors:** David J. Whiteside, Maura Malpetti, P. Simon Jones, Boyd C. P. Ghosh, Ian Coyle-Gilchrist, John C. van Swieten, Harro Seelaar, Lize Jiskoot, Barbara Borroni, Raquel Sanchez-Valle, Fermin Moreno, Robert Laforce, Caroline Graff, Matthis Synofzik, Daniela Galimberti, Mario Masellis, Maria Carmela Tartaglia, Elizabeth Finger, Rik Vandenberghe, Alexandre de Mendonça, Fabrizio Tagliavini, Chris R. Butler, Isabel Santana, Isabelle Le Ber, Alexander Gerhard, Simon Ducharme, Johannes Levin, Adrian Danek, Markus Otto, Sandro Sorbi, Florence Pasquier, Arabella Bouzigues, Lucy L. Russell, Jonathan D. Rohrer, James B. Rowe, Timothy Rittman

**Affiliations:** 1 Department of Clinical Neurosciences, University of Cambridge, Cambridge, Cambridgeshire, UK; 2 Cambridge University Hospitals NHS Foundation Trust, Cambridge, UK; 3 Wessex Neurological Centre, University Hospital Southampton, Southampton, UK; 4 Norfolk and Norwich University Hospital, Norwich, UK; 5 Department of Neurology, Erasmus Medical Centre, Rotterdam, Netherlands; 6 Centre for Neurodegenerative Disorders, Department of Clinical and Experimental Sciences, University of Brescia, Brescia, Italy; 7 Alzheimer’s Disease and Other Cognitive Disorders Unit, Neurology Service, Hospital Clinic, Institutd’Investigacións Biomèdiques August Pi I Sunyer, University of Barcelona, Barcelona, Spain; 8 Cognitive Disorders Unit, Department of Neurology, Donostia University Hospital, San Sebastian, Gipuzkoa, Spain; 9 Neuroscience Area, Biodonostia Health Research Institute, San Sebastian, Gipuzkoa, Spain; 10 CHU de Québec, and Faculté de Médecine, Département des Sciences Neurologiques, Clinique Interdisciplinaire de Mémoire, Université Laval, QC, Canada; 11 Center for Alzheimer Research, Division of Neurogeriatrics, Department of Neurobiology, Care Sciences and Society, Bioclinicum, Karolinska Institutet, Solna, Sweden; 12 Unit for Hereditary Dementias, Theme Aging, Karolinska University Hospital, Solna, Sweden; 13 Department of Neurodegenerative Diseases, Hertie-Institute for Clinical Brain Research, Tübingen, Germany; 14 Center of Neurology, University of Tübingen, Tübingen, Germany; 15 Fondazione IRCCS Ospedale Policlinico, Milan, Italy; 16 Department of Biomedical, Surgical and Dental Sciences, University of Milan, Milan, Italy; 17 Sunnybrook Health Sciences Centre, Sunnybrook Research Institute, University of Toronto, Toronto, Canada; 18 Tanz Centre for Research in Neurodegenerative Diseases, University of Toronto, Toronto, Canada; 19 Department of Clinical Neurological Sciences, University of Western Ontario, London, Ontario, Canada; 20 Laboratory for Cognitive Neurology, Department of Neurosciences, KU Leuven, Leuven, Belgium; 21 Neurology Service, University Hospitals Leuven, Belgium; 22 Leuven Brain Institute, KU Leuven, Leuven, Belgium; 23 Faculty of Medicine, University of Lisbon, Lisbon, Portugal; 24 Fondazione IRCCS Istituto Neurologico Carlo Besta, Milano, Italy; 25 Nuffield Department of Clinical Neurosciences, Medical Sciences Division, University of Oxford, Oxford, UK; 26 Department of Brain Sciences, Imperial College London, London, UK; 27 University Hospital of Coimbra (HUC), NeurologyService, Facultyof Medicine, Universityof Coimbra, Coimbra, Portugal; 28 Center for Neuroscience and Cell Biology, Faculty of Medicine, University of Coimbra, Coimbra, Portugal; 29 Paris Brain Institute – Institut du Cerveau – ICM, Inserm U1127, CNRS UMR7225, AP-HP- Hôpital Pitié-Salpêtrière, Sorbonne Università, Paris, France; 30 Centrede référence des démences rares ou précoces, IM2A, Département de Neurologie, AP-HP- Hôpital Pitié-Salpêtrière, Paris, France; 31 Départementde Neurologie, AP-HP- Hôpital Pitié-Salpêtrière, Paris, France; 32 Division of Neuroscience and Experimental Psychology, Wolfson Molecular Imaging Centre, University of Manchester, Manchester, UK; 33 Departments of Geriatric Medicine and Nuclear Medicine, University of Duisburg- Essen, Duisburg, Germany; 34 Department of Psychiatry, McGill University Health Centre, McGill University, Montreal, Québec, Canada; 35 Department of Neurology & Neurosurgery, McConnell Brain Imaging Centre, Montreal Neurological Institute, McGill University, Montreal, Canada; 36 Neurologische Klinik, Ludwig-Maximilians-Universität München, Munich, Germany; 37 German Center for Neurodegenerative Diseases (DZNE), Munich, Germany; 38 Munich Cluster of Systems Neurology, Munich, Germany; 39 Department of Neurology, University of Ulm, Ulm, Germany; 40 Department of Neurofarba, University of Florence, Florence, Italy; 41 IRCCS Fondazione Don Carlo Gnocchi, Florence, Italy; 42 Univ Lille, Lille, France; 43 Inserm 1172, Lille, France; 44 CHU, CNR-MAJ, Labex Distalz, LiCEND Lille, Lille, France; 45 Department of Neurodegenerative Disease, Dementia Research Centre UCL Institute of Neurology, Queen Square, London, UK; 46 MRC Cognition and Brain Sciences Unit, University of Cambridge, Cambridge, UK

**Keywords:** disease progression, frontotemporal dementia, functional magnetic resonance imaging (fMRI), network dynamics, presymptomatic

## Abstract

**Introduction:**

We tested whether changes in functional networks predict cognitive decline and conversion from the presymptomatic prodrome to symptomatic disease in familial frontotemporal dementia (FTD).

**Methods:**

For hypothesis generation, 36 participants with behavioral variant FTD (bvFTD) and 34 controls were recruited from one site. For hypothesis testing, we studied 198 symptomatic FTD mutation carriers, 341 presymptomatic mutation carriers, and 329 family members without mutations. We compared functional network dynamics between groups, with clinical severity and with longitudinal clinical progression.

**Results:**

We identified a characteristic pattern of dynamic network changes in FTD, which correlated with neuropsychological impairment. Among presymptomatic mutation carriers, this pattern of network dynamics was found to a greater extent in those who subsequently converted to the symptomatic phase. Baseline network dynamic changes predicted future cognitive decline in symptomatic participants and older presymptomatic participants.

**Discussion:**

Dynamic network abnormalities in FTD predict cognitive decline and symptomatic conversion.

## 1 Introduction

Neuropathological and structural changes accumulate over many years prior to the onset of symptoms in neurodegenerative diseases.^1,2^ Understanding the timing and consequence of such changes for clinical syndromes is key to accounting for heterogeneity in progression and risk-stratifying asymptomatic individuals for preventative clinical trials. We have previously shown that functional network integrity is important in maintaining cognitive performance in individuals at risk of dementia,^3,4^ with the corollary that loss of network integrity may herald symptom onset and predict cognitive decline. Genetic frontotemporal dementia (FTD) provides an opportunity to characterize functional networks throughout the course of the disease. Approximately one-third of patients with FTD have a family history in keeping with an autosomal dominant inheritance.^5^ Mutations in three genes account for the majority of these cases: chromosome 9 open reading frame 72 (C9orf72), granulin (GRN), and microtubule- associated protein tau (MAPT).^5,6^ The resulting phenotypes are heterogeneous, with behavioral variant FTD (bvFTD) the most common clinical presentation.^5^

The coordination of neural activity across distributed spatial and temporal scales is dynamic.^7–9^ Such connectivity underpins cognition in health and is affected in psychiatric and neurodegenerative diseases.^10–12^ While canonical approaches to functional connectivity have averaged over the scan acquisition time, time-varying fluctuations in connectivity can also be captured by functional magnetic resonance imaging (fMRI).^13–15^ In the clinical syndromes of FTD, there are deficits in inhibitory and excitatory neurotransmitters required for network integration and segregation^16^ which we propose contribute to changes in temporal dynamics in the disease. Subtle changes in time-varying functional connectivity occur in presymptomatic mutation carriers,^17^ although their longitudinal significance and evolution into the symptomatic phase have not been studied.

We examined resting state brain dynamics in presymptomatic and symptomatic carriers of pathogenic mutation carriers in the Genetic Frontotemporal Initiative (GENFI) using fMRI to determine whether disruption to network dynamics predicts cognitive decline. We used hidden Markov modelling as a highly articulated data-driven approach to model the blood-oxygen-level-dependent signal of fMRI, an approach which posits the existence of a finite number of hidden states that describe the sequential evolution of observed data.^15,18^

We investigated brain state dynamics using hidden Markov models (HMMs) with a two-stage approach to ensure replication and refine analytic choices. Hypothesis generation used a cohort of patients with mainly sporadic bvFTD and control participants recruited at the Cambridge Centre for FTD. We repeated the methodology in the GENFI, following preregistration of our cross-sectional analysis plan (https://osf.io/k64gh/wiki/home/), with the following hypotheses: (1) brain state dynamics differ between symptomatic mutation carriers and cognitively normal non-mutation carriers; (2) changes in network dynamics correlate with both neuropsychological deficits and carer assessed measures of impairment; (3) presymptomatic mutation carriers (versus non-mutation carriers) have abnormal network dynamics as a function of proximity to onset as denoted by age; and (4) altered network dynamics predict conversion from the presymptomatic to symptomatic phase and subsequent cognitive decline in gene mutation carriers.

## 2 Materials And Methods

### 2.1 Participants

We used datasets from 36 participants with bvFTD and 34 healthy controls recruited at the Cambridge University Centre for Frontotemporal Dementia for hypothesis generation. Clinical assessment included the Addenbrooke’s Cognitive Examination-Revised,^19^ Mini-Mental State Examination (MMSE),^20^ Frontal Assessment Battery,^21^ and Cambridge Behavioral Inventory-Revised (CBI-R).^22^

The GENFI includes participants from 25 research sites across Europe and Canada. Participants were included if they were over 18 and had a known pathogenic mutation in MAPT, C9ORF72, GRN, or TBK1, or were a first degree relative of a mutation carrier. A total of 198 symptomatic mutation carriers, 341 asymptomatic mutation carriers, and 329 family members with usable fMRI (datafreeze 5) were included in this study. Clinicians classified mutation carriers as either symptomatic or presymptomatic, with participants deemed symptomatic if symptoms were present, were progressive in nature, and consistent with a diagnosis of an FTD-related degenerative disorder.

GENFI participants underwent a standardized assessment with clinical history, physical examination, neuropsychological assessment, and informant-based questionnaires.^1^ Severity of behavioral symptoms was assessed using the CBI-R. Neuropsychological tests included the Trail Making Tests, Digit Symbol Test, Backwards Digit Span, Letter and Category Fluency, a short version of the Boston Naming Test,^23^ and the MMSE. Assessments were repeated yearly or biannually, with longitudinal data up to 7 years post baseline visit.

#### 2.2 Image acquisition and preprocessing

Image acquisition for the two cohorts and fMRI preprocessing have been published previously^3,4,24^ and are described in detail in the Supplementary Materials. Given the potential sensitivity of estimates of network dynamics to motion,^25,26^ we excluded participants above previously defined thresholds for three data quality indices^24^ (maximum spike percentage,^27^ maximum framewise displacement,^25^ maximum spatial standard deviation of successive volume difference^28^). We excluded nine participants with bvFTD and 2 healthy controls from the Cambridge cohort, and 103 scans from 89 participants in the GENFI (20 non-carriers, 21 presymptomatic mutation carriers, 48 symptomatic participants). We performed an additional analysis excluding participants exceeding a mean framewise displacement threshold but included in the primary analysis (Supplementary Materials).

#### 2.3 Hidden Markov models (HMMs)

We assessed network dynamics in both cohorts using HMMs.^29^ These models treat time series data as being generated from a finite number of unknown states. Each time point is therefore classified as being in a single state, although the assumption of state mutual exclusivity is adjusted through soft probabilistic inference. While the states and probability of transitioning between them are defined at the group level, a state time course can be estimated per participant.

We performed an independent component analysis (ICA) using MELODIC (fMRIB Software Library [FSL]) from preprocessed fMRI of all participants to allow comparison between cohorts. We chose a model order of 30 as a balance between excessive network fragmentation^30^ and predetermining HMM outputs. Six component maps were identified as artefact. Participant specific time courses for each component were generated by regression of the component maps into each subject’s preprocessed fMRI. From standardized per participant non-artefactual component time courses a multivariate Gaussian HMM^15^ with six brain states was inferred for each cohort using the HMM-MAR toolbox (https://github.com/OHBA-analysis/HMM-MAR). All states shared a common covariance matrix.^31^ Model order was specified in registration prior to analyzing the GENFI dataset; it has previously been shown that robust behavioral inferences can be made through a six-state model.^32^ We repeated the analysis with ICA dimensionality determined automatically by MELODIC.

The temporal dynamics of HMM states can be characterized through a small set of metrics, namely switching rate (the frequency with which state transitions occur), fractional occupancy (the proportion of time a state is active), and the transition matrix consisting of transition probabilities (the chance of between-state transitions) and persistence probabilities (the chance of remaining in the same state). Mean activation maps were generated by weighting component maps by the mean of the state Gaussian distribution. For illustrative purposes we compared these maps with templates maps of canonical static functional networks,^33^ and performed an additional analysis of resting state connectivity to determine where connectivity changes occur in the GENFI cohort (see Supplementary Materials).

### 2.4 Statistical analyses

All statistical analyses were performed in R,^34^ with the exception of permutation testing using FSL’s PALM (“Permutation Analysis of Linear Models”).^35^ *P*-values throughout were corrected across relevant tests for a false discovery rate of 5%, except permutation testing where family-wise error correction to 5% was performed across all tests and contrasts.

#### 2.4.1 Descriptive statistics

We compared continuous variables between groups using independent sample *t* tests and categorical variables with the chi-square test.

#### 2.4.2 Cambridge cohort

We compared fractional occupancy and switching rates between groups using a one-way analysis of covariance, with age and sex as covariates. For each participant we extracted matrices of the 36 transition and persistence probabilities. Given the interdependence of these probabilities, we assessed for group differences in a permutation test (5000 permutations) using FSL’s PALM. Age and sex were included as covariates of no interest.

#### 2.4.3 GENFI

In the GENFI data, cross-sectional analysis was performed using participants’ latest scan that passed motion thresholding, maximizing per-participant volume number. Differences in fractional occupancy rates and switching rates were assessed using mixed-effects linear models with diagnostic group as the main effect, age and sex as dependent variables, and scan site as a random intercept using the lme4 package.^36^ Significance values were calculated using the Satter-waithe estimate of effective degrees of freedom. Switching rates were adjusted to account for small differences in repetition time. Group differences in transition/persistence probabilities were calculated as per the Cambridge cohort.

For contrasts with clinical scores and longitudinal analysis we performed a principal component analysis on state fractional occupancies using the *alfa.pca* (alpha = 1) function from the Compositional package in R,^37^ followed by varimax rotation. We selected the number of components for analysis using MacArthur’s “broken-stick” criterion.^38^

#### 2.4.4 Network dynamics by age

In previous GENFI studies, mean family age at symptom onset has been used to estimate years until symptom onset, but only in MAPT mutations does this explain a significant proportion of variability in age of onset.^39^ Given that component scores did not differ by mutation type, we explored component scores by age as a marker of proximity to onset (comparing to family members without mutations, over a similar age range). We compared component scores and state occupancies between non-carriers and presymptomatic mutation carriers as a function of age, assessing the group by age (linear or quadratic) interaction.

#### 2.4.5 Presymptomatic mutation carriers and neuropsychological correlates

We compared component scores in presymptomatic mutation carriers with pre-registered neuropsychological tests (Backwards Digit Span, Digit Symbol, Trail Making Test) as a function of age within a mixed-effects linear model.

#### 2.4.6 Converters

Mutation carriers who were assessed during longitudinal follow-up as moving from the presymptomatic to symptomatic phase were classified as *converters*. We compared component scores, state occupancies, and neuropsychological scores between converters and nonconverting presymptomatic mutation carriers at their latest presymptomatic scan in mixed-effects linear models with group as the main effect, age and sex as covariates, and scan site as a random variable.

#### 2.4.7 Longitudinal cognitive data in symptomatic patients

A mixed linear model was used to calculate patient specific yearly rates of change in clinical and neurocognitive scores (MMSE, CBI-R, Backwards Digit Span, Digit Symbol, Trail Making Test B [TMTB]). Neurocognitive assessment was the dependent variable in the model, with years from baseline assessment as an independent variable and with estimation of intercept and slope (neurocognitive assessment ~ time + (time | ID)). These models were calculated using all participants.

To assess whether baseline component scores predict neurocognitive decline, individual estimates of disease progression (slope) were taken to a second model as a dependent variable, with baseline component scores as an independent variable and baseline age, sex, and site as covariates of no interest.

#### 2.4.8 Longitudinal cognition in presymptomatic mutation carriers

We repeated the two-step model for presymptomatic mutation carriers, additionally assessing the interaction between baseline component scores and age given that proximity to symptom onset increases the probability that small fluctuations in neurocognitive assessment are important.

## 3 Results

### 3.1 Descriptive statistics

Demographic and clinical characteristics for the two cohorts for participants with a sub-motion threshold scan are included in Tables 1 and 2.

In the Cambridge cohort no significant differences were observed in age or sex. In GENFI, symptomatic participants were older than asymptomatic participants and showed marked deficits in neuropsychological and informant-based assessment of severity.

### 3.2 Network dynamics in FTD

For the Cambridge data, we used temporally concatenated participant time series from ICA components to fit an HMM with six brain states (Figure S1A, with labeling in Figure S2 to indicate the most closely matching canonical static network for positive and negative activations). Participants with FTD had increased fractional occupancy of state 2, whose positive activations constituted the salience network (*F* = 7.8, *P* = .043). Switching rates between states were reduced in FTD (Figure S1; *F* = 6.5, *P* = .014).

**Figure 1:**
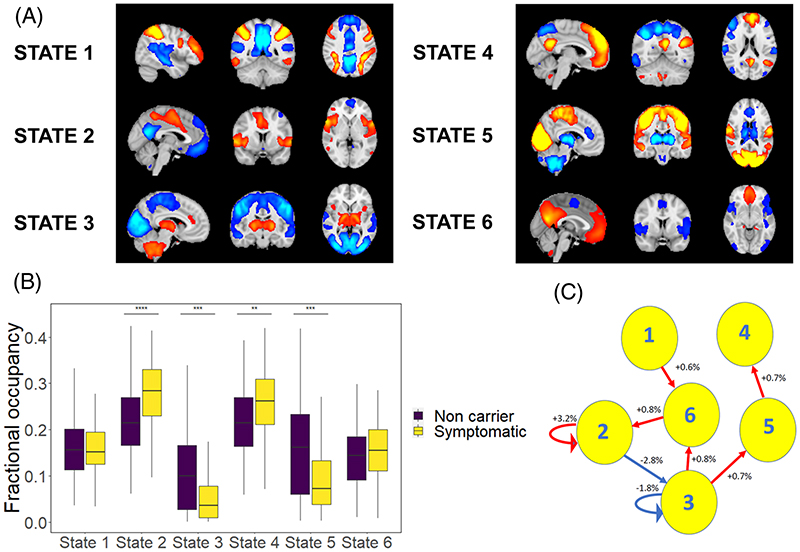
Network dynamics in the Genetic Frontotemporal Initiative. (A) Mean activation maps for the six modelled states. (B) Fractional occupancy by state, with increased occupancy in states 2 and 4, and decreased occupancy in states 3 and 5. (C) Altered transition and persistence probabilities in frontotemporal dementia (FTD) using a permutation test. Blue lines represent significantly decreased transitions in FTD, and red lines significantly increased transitions. The figures show the absolute percentage increase or decrease in probability in FTD

For the GENFI data, we used temporally concatenated participant component timeseries to fit an HMM with six brain states (Figure 1A-C and S2). Comparing symptomatic participants with mutation noncarriers, we found that participants with FTD had increased fractional occupancy of the state overlapping with the salience network (state 2, *F* = 32, corrected *P* = 2 × 10^−7^) and of state 4 overlapping with the default mode network (*F* = 8, *P* = .008). Participants with FTD spent less time than non-carriers in two states with inversed activation patterns: state 3 with positive activations in sub-cortical regions (*F* = 17, *P* = 1 × 10^−4^), and state 5 with positive activations in motor and sensory (somatic, visual, and auditory) regions (*F* = 15, corrected *P* = 2 × 10^−4^). In this cohort switching rates did not differ in FTD (*F* = 3.1, *P* = .08).

**Figure 2:**
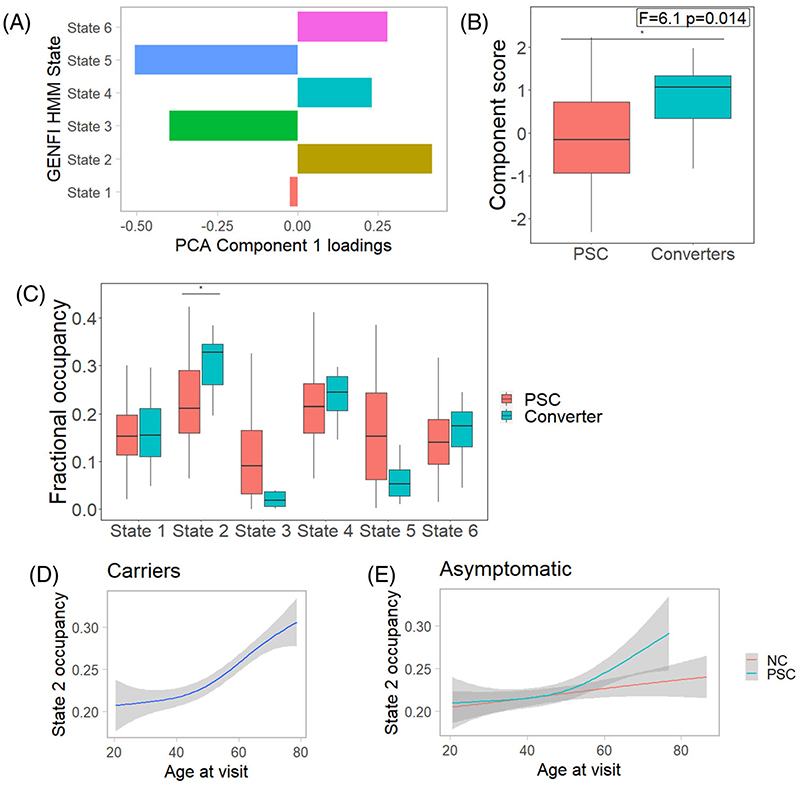
Changes in network dynamics occurring in the late presymptomatic phase. (A) Component loadings from a principal component analysis (PCA) on state occupancies. (B) Component scores showing a significant increase in converters (at their latest presymptomatic scan) in contrast to those who have not converted to the symptomatic phase during longitudinal follow-up. (C) Fractional occupancy by state, showing an increase for converters in state 2 (salience) occupancy. (D) State 2 occupancy in all carriers. (E) State 2 occupancy in presymptomatic mutation carriers (PSC) showing evidence of a non-linear relationship with age, in contrast to non-carriers (NC). GENFI, Genetic Frontotemporal Initiative; HMM, hidden Markov model

We performed a principal component analysis with varimax rotation on state occupancies for each cohort. In the GENFI cohort one component was selected, which explained 68% of the variance (Figure 2A). Higher component scores were associated with greater time in states 2, 4, and 6, and decreased time in states 3 and 5. Component scores were increased in symptomatic participants (*F* = 21, *P* = 4 × 10^−7^). There was a weak trend between component scores and motion assessment indices in symptomatic participants (maximum framewise displacement Pearson’s *R* = 0.047, *P* = .57; maximum DVARS *R* = 0.042, *P* = .61; maximum spike percentage *R* = 0.1 *P* = .1). Comparable components were derived for the Cambridge cohort (Supplementary Materials).

We found no difference in component scores by mutation or clinical phenotype (Supplementary Materials). Component scores were associated with carer-based assessments and neuropsychological scores in symptomatic and presymptomatic mutation carriers (Supplementary Materials and Figure S4).

### 3.5 Network dynamics in mutation carriers

We investigated temporal dynamics across all mutation carriers. We hypothesized that fractional occupancy would show a non-linear relationship with age, as a proxy marker of proximity to symptom onset. We therefore included a quadratic term for age using orthogonalized polynomials. Model comparison found that inclusion of a quadratic age term to a linear model significantly improved fit for state 2, but not for component scores or other states (Table S2 and Figure 2D).

Within a mixed model including age as a quadratic term and with sex and site as covariates of no interest, state 2 occupancy showed an uncorrected difference between non-carriers and presymptomatic mutation carriers as a function of age (interaction *F* = 3.8, uncorrected *P* = .022, Figure 2E), results that were not replicated in a purely linear model (*F* = 1.7, uncorrected *P* = .19). No differences were observed for other states or components scores.

### 3.4 Network dynamics predict symptomatic conversion

Fourteen presymptomatic carriers became symptomatic during follow-up. We compared these converters at their latest presymptomatic visit with imaging with other presymptomatic carriers. Converters had significantly worse performance on neuropsychological assessment at this visit (Backwards Digit Span *F* = 5.7, *P* = .017; Backwards Digit Span score *F* = 6.9, *P* = .009; Trail Making Test B *F* = 28, *P* = 2 × 10^−7^). We found that component scores and state 2 occupancy were increased in converters (Figure 2B,C).

### 3.5 Network dynamics predict cognitive decline

We assessed whether higher baseline component scores in symptomatic patients were associated with subsequent neurocognitive decline using pre-registered assessments (TMTB, Digit Symbol, Backwards Digit Span) and measures of global cognitive and behavioral decline (CBI-R, MMSE). Patients at floor scores for assessments were removed prior to deriving linear mixed models (TMTB *n* = 20, Backwards Digit Span *n* = 2, Digit Symbol *n* = 2). Linear mixed models on longitudinal clinical and neurocognitive scores indicated an effect of time for all measures in symptomatic participants (Table S3).

**Figure 3:**
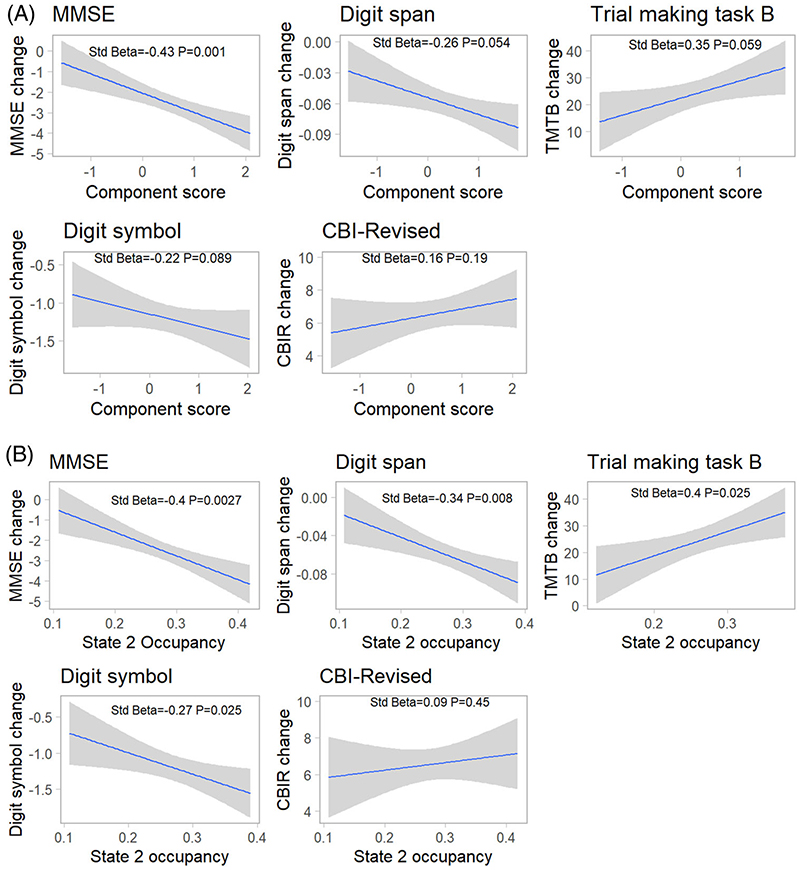
Cognitive decline in symptomatic participants. (A) Baseline component scores predict subsequent cognitive decline in symptomatic participants in the Mini-Mental State Examination (MMSE), with an uncorrected association with Digit Span and Trail Making Test B (TMTB). Annualized rates of change in cognitive scores are derived from a mixed linear effect model, and taken to a second model to compare with component scores while partialing out covariates. (B) Baseline state 2 occupancy predicts subsequent cognitive decline in symptomatic patients in a range of clinical and neuropsychological tests. CBI-R, Cambridge Behavioral Inventory-Revised

Correcting for age at baseline scan, sex, and site, baseline component scores were related to the annual rate of clinical progression for MMSE (Figure 3A, Std Beta = −0.43, *P* = .001). The associations with Backwards Digit Span (Std Beta = −0.26, uncorrected *P* = .021, *P* = .054) and TMTB (Std Beta = 0.35, uncorrected *P* = .035, *P* = .059) were not significant after correction for multiple comparisons. No significant relationship was found with Digit Symbol (Std Beta = −0.21, *P* = .089) or carer-rated severity using the CBI-R (Std Beta = 0.16 *P* = .18). We found a significant difference in slope between symptomatic mutation carriers and non-carriers for MMSE, TMTB, and CBI-R (group × baseline component score interaction: MMSE Std Beta = −0.66, *P* = 2 × 10^−10^; Backwards Digit Span Std Beta = −0.23 *P* = .11; Digit Symbol Std Beta = −0.12 *P* = .18; TMTB Std Beta = 0.58 *P* = 5 × 10^−5^; CBI-RStd Beta = 0.12 *P* = .041).

We proceeded to investigate whether baseline network dynamics predicted cognitive and clinical decline in presymptomatic mutation carriers, hypothesizing that the relationship between annualized rate of change in neurocognitive measure and component scores would depend on age as a marker of proximity to symptom onset.

We found that age significantly modified the relationship between annualized rate of clinical progression and baseline component scores for TMTB (interaction Std Beta = 0.21 *P* = .002), and MMSE (interaction Std Beta = −0.14 *P* = .048). For the TMTB, a significant three-way interaction (group × age × component score) implied that baseline component score increased the rate of clinical deterioration in older presymptomatic mutation carriers, relative to non-carriers or younger carriers (Table 3). We did not find any significant relationships with Digit Symbol, Backwards Digit Span, or CBI-R.

Given the difference in state 2 occupancies both in converters and between non-carriers and presymptomatic mutation carriers, together with the known role of the salience network in FTD, we also investigated the relationship between baseline state 2 occupancy and longitudinal cognitive decline. We found baseline salience state occupancy predicted cognitive decline in symptomatic carriers in all measures except CBI-R, and for the TMTB, MMSE, and CBI-R in older presymptomatic mutation carriers (Figure 3B and supplementary materials).

## 4 Discussion

This study demonstrates that the temporal dynamics of large-scale brain networks are disrupted by sporadic and familial FTD, with characteristic changes in both the symptomatic and late presymptomatic phases of disease. There is an increase in salience and default mode network occupancy, and a decrease in proportion of time spent in the primary cortices and subcortical regions: a change which correlates with clinical and neuropsychological markers of disease severity. Changes in temporal dynamics occur near to disease onset and predict the onset and deterioration of the clinical syndrome as evidenced by (1) the increased component scores of those who subsequently converted to the symptomatic phase during follow-up, and (2) increased rates of cognitive and clinical decline in both symptomatic and older presymptomatic participants with higher component scores.

Functional networks provide an intermediate phenotype to investigate the compensatory changes that account for the dissociation between neuropathological progression and maintained cognitive performance in presymptomatic neurodegeneration,^40^ with coupling between functional connectivity and cognition increasing close to disease onset.^4,41^ Changes in time-varying connectivity predict behavioral traits beyond static functional connectivity or structure alone,^11,31^ suggesting that investigating network dynamics informs our understanding of the transition from the presymptomatic to symptomatic phase of neurodegenerative disease. Here we found that while the dynamic repertoire is unchanged through much of the presymptomatic period, the onset of change indicates future symptomatic decline. This suggests that network dynamics can potentially be used both to guide prognosis and as an intermediate marker of success for interventions in presymptomatic mutation carriers, adding to existing clinical, blood, and other imaging biomarkers.^42^

Given that the salience network is selectively targeted in bvFTD, with atrophy of network hubs and reduced functional connectivity,^43–45^ the finding of increased salience network occupancy in FTD in both cohorts is perhaps unexpected. The salience network is integral to accessing other large-scale networks, including executive^46^ and default mode networks.^47^ Neuropathological disruption to salience network connectivity may undermine its ability to coordinate network switching, perturbing global network dynamics, resulting in increased time spent in a state with positive activations in the default mode network and increased time within the salience network itself. Assessment of between-group differences in transition probabilities provides a potential explanation for these changes. We found a reduced frequency of transition from the salience state to the subcortical (primarily thalamic) state. Subcortical atrophy is well recognized in FTD, notably in the thalamus, and occurs in both sporadic and genetic FTD,^48,49^ including in the presymptomatic phase.^1^ Our findings could suggest that subcortical network integrity influences cortical salience network dynamics, echoing previous work describing the role of thalamic degeneration in disrupting salience network connectivity in genetic FTD.^48^

There are limitations to our study, despite the advantages of crosssectional replication and longitudinal follow-up in the GENFI data. The HMM provides a data-driven explanation of the data without biological assumptions,^50^ with resulting constraints to its explanatory power. It is possible that a time-varying connectivity approach with additional biologically informed constraints could provide further group differentiation and refined longitudinal predictions. Our approach was not optimized to find differences in brain state dynamics between mutation types or by phenotype. Alternative methodological choices may reveal such differences, according to different a priori numbers of states, focusing on different large-scale networks and modelling subsets of patients. In the GENFI cohort the study design necessarily results in a significant age difference between non-carriers and symptomatic participants. That similar patterns of state occupancies were observed in the Cambridge cohort suggests that our results are not primarily driven by age differences.

We conclude that network dynamics are a critical link between neuropathology and symptomatology, heralding symptom onset and correlating with key measures of clinical severity. Network dynamics are a promising tool for stratification and prognostication in FTD.

## Supplementary Material

Supplementary Material

Appendix

## Figures and Tables

**Table 1:** Demographic and clinical characteristics for the participants recruited at the Cambridge Centre for Frontotemporal Dementia and Related Disorders

	Control (*n* = 32)	FTD (*n* = 27)	Statistic (*t*/χ^2^)
Age	67.2 (8.5)	64.3 (7.3)	*t*(57) = 1.4, *P* = .16
Sex (M/F)	14/18	17/11	*χ* = 1.5, *P=* .23
ACE-R		65 (20)	
FAB		9.3 (4.4)	
CBI-R		74.3(22.3)	

Scores are mean (SD). ACE-R, Addenbrookes Cognitive Examination-Revised; CBI-R, Cambridge Behavioral Inventory-Revised; FAB, Frontal Assessment Battery.

**Table 2:** Demographic and clinical characteristics for the GENFI participants

	NC	PSC	Symp	NC vs. Symp			NCvs. PSC		
	*n* = 309	*n* = 320	*n* = 150	Stat (*χ*2/*t*)	*P*	*d*	Stat (*χ*2/*t*)	*P*	*d*
Age (y)	48 (13)	45 (12)	63 (8.2)	*t* = −15	<.0001	1.3	*t* = 2.5	.01	0.2
Gender (F/M)	179/130	197/123	67/83	X = 7	0.008		X = 0.9	0.35	
Mutation (n)	C9orf72 109 GRN 133 MAPT 60 TBK1 7	C9orf72 119 GRN 141 MAPT 58 TBK1 2	C9orf72 71 GRN 53 MAPT 26	X = 5	.06		X = 0.3	.9	
MMSE	29 (1)	29 (1)	21 (7)	*t* = 13	<.0001	1.9	*t* = −0.1	.92	0
CBI-RTotal	5 (7)	6 (9)	62 (32)	*t* = −21	<.0001	−3	*t* = −1.5	.1	−0.13
Trail Making Test B	67 (37)	67 (40)	211 (92)	*t* = −16	<.0001	-2.4	*t* = 0.13	.99	0
Digit Symbol	58 (14)	58 (14)	25 (14)	*t* = 22	<.0001	2.3	*t* =0	1	0
Backwards Digit Span score	4.8(1.2)	4.8 (1.2)	3.1 (1.5)	*t* = 13	<.0001	1.4	*t* = −0.54	.6	-0.04
Boston Naming	28 (2)	28 (3)	19 (8)	*t* = 13	<.0001	1.8	*t* = 0.84	.4	0.07
Letter Fluency	41 (13)	41 (13)	17 (12)	*t* = 18	<.0001	1.9	*t* = 0.84	.4	0.07
Category Fluency	23 (6)	24 (6)	11 (6)	*t* = 20	<.0001	2.2	*t* = −1.5	.14	−0.12

Scores are mean (SD). *P* values minimum threshold of <.0001. CBI-R, Cambridge Behavioral Inventory-Revised; MMSE, Mini-Mental State Examination; NC, non-carrier; PSC, presymptomatic mutation carrier; Symp, symptomatic.

**Table 3:** Two-step prediction models for presymptomatic mutation carriers

Model	Slope ~ comp + cov			Slope ~ comp*age + cov			Slope ~ comp*age*group + cov		
	*Std Beta*	*t*	*P*	*Std Beta*	*t*	*P*	*Std Beta*	*t*	*P*
TMTB	−0.13	−1.0	.75	0.22	3.9	.0006	0.43	5.1	2x10^-6^
Digit Span	0.02	0.37	.75	0.09	1.5	.17	0.12	1.3	.19
Digit Symbol	−0.02	−0.31	.75	−0.02	−0.45	.66	−0.12	−1.6	.15
MMSE	−0.05	−0.76	.75	−0.14	−2.4	.048	−0.19	−2.1	.072
CBI-R	0.03	0.50	.75	0.11	1.7	.15	0.20	2.0	.072

comp, fractional occupancy component; CBI-R, Cambridge Behavioral Inventory-Revised; MMSE, Mini-Mental State Examination; TMTB, Trail Making Test B.

## Highlight

1. We investigated brain network predictors of dementia symptom onset
2. Frontotemporal dementia results in characteristic dynamic network patterns
3. Alterations in network dynamics are associated with neuropsychological impairment
4. Network dynamic changes predict symptomatic conversion in presymptomatic carriers
5. Network dynamic changes are associated with longitudinal cognitive decline

## Research In Context

### 1 Systematic Review

We reviewed published literature using traditional resources. Neuropathological and structural changes occur in dementia many years prior to the onset of symptoms. Assessing the onset of the symptomatic phase in those at high risk of dementia is clinically challenging, and neural correlates of conversion from the presymptomatic prodrome to symptomatic disease are not well characterized.

### 2 Interpretation

Our results show that changes in the proportion of time spent in key brain networks in presymp-tomatic carriers of frontotemporal dementia mutations occur in the late presymptomatic phase. They are associated with conversion to symptomatic disease and subsequent cognitive decline.

### 3 Future Directions

Dynamic brain network changes are a promising tool for stratification and prognostication in presymptomatic dementia, with implications for predicting outcome and risk-stratifying asymptomatic individuals for preventative clinical trials

## References

[1] Rohrer JD, Nicholas JM, Cash DM (2015). Presymptomatic cognitive and neuroanatomical changes in genetic frontotemporal dementia in the Genetic Frontotemporal dementia Initiative (GENFI) study: a cross-sectional analysis. Lancet Neuro.

[2] Oxtoby NP, Young AL, Cash DM (2018). Data-driven models of dominantly-inherited Alzheimer’s disease progression. Brain.

[3] Rittman T, Borchert R, Jones S (2019). Functional network resilience to pathology in presymptomatic genetic frontotemporal dementia. Neurobiol Aging.

[4] Tsvetanov KA, Gazzina S, Jones PS (2020). Brain functional network integrity sustains cognitive function despite atrophy in presymp-tomatic genetic frontotemporal dementia. Alzheimers Dement.

[5] Greaves CV, Rohrer JD (2019). An update on genetic frontotemporal dementia. J Neurol.

[6] Rohrer JD, Guerreiro R, Vandrovcova J (2009). The heritability and genetics of frontotemporal lobar degeneration. Neurology.

[7] Tognoli E, Kelso JAS (2014). The metastable brain. Neuron.

[8] Breakspear M (2017). Dynamic models of large-scale brain activity. Nat Neurosci.

[9] Shine JM, Breakspear M, Bell PT (2019). Human cognition involves the dynamic integration of neural activity and neuromodulatory systems. Nat Neurosci.

[10] Filippi M, Spinelli EG, Cividini C, Agosta F (2019). Resting state dynamic functional connectivity in neurodegenerative conditions: a review of magnetic resonance imaging findings. Front Neurosci.

[11] Liégeois R, Li J, Kong R (2019). Resting brain dynamics at different timescales capture distinct aspects of human behavior. Nat Commun.

[12] Fu Z, Iraji A, Turner JA (2021). Dynamic state with covarying brain activity-connectivity: on the pathophysiology of schizophrenia. Neuroimage.

[13] Chang C, Glover GH (2010). Time-frequency dynamics of resting-state brain connectivity measured with fMRI. Neuroimage.

[14] Calhoun VD, Miller R, Pearlson G, Adali T (2014). The chronnectome: time-varying connectivity networks as the next frontier in fMRI data discovery. Neuron.

[15] Vidaurre D, Smith SM, Woolrich MW (2017). Brain network dynamics are hierarchically organized in time. Proc Natl Acad Sci.

[16] Murley AG, Rowe JB (2018). Neurotransmitter deficits from frontotemporal lobar degeneration. Brain.

[17] Premi E, Calhoun VD, Diano M (2019). The inner fluctuations of the brain in presymptomatic frontotemporal dementia: the chronnectome fingerprint. Neuroimage.

[18] Vidaurre D, Abeysuriya R, Becker R (2018). Discovering dynamic brain networks from big data in rest and task. Neuroimage.

[19] Mioshi E, Dawson K, Mitchell J, Arnold R, Hodges JR (2006). The Adden-brooke’s Cognitive Examination Revised (ACE-R): a brief cognitive test battery for dementia screening. Int J Geriat Psychiatry.

[20] Folstein MF, Folstein SE, McHugh PR (1975). Mini-mental state. J Psychiatr Res.

[21] Dubois B, Slachevsky A, Litvan I, Pillon B (2000). The FAB: a frontal assessment battery at bedside. Neurology.

[22] Wear HJ, Wedderburn CJ, Mioshi E (2008). The Cambridge behavioural inventory revised. Dement Neuropsychol.

[23] Morris JC, Weintraub S, Chui HC (2006). The Uniform Data Set (UDS): clinical and cognitive variables and descriptive data from Alzheimer disease centers. Alzheimer Dis Assoc Disord.

[24] Whiteside DJ, Jones PS, Ghosh BCP (2021). Altered network stability in progressive supranuclear palsy. Neurobiol Aging.

[25] Power JD, Barnes KA, Snyder AZ, Schlaggar BL, Petersen SE (2012). Spurious but systematic correlations in functional connectivity MRI networks arise from subject motion. Neuroimage.

[26] Laumann TO, Snyder AZ, Mitra A (2017). On the stability of BOLD fMRI Correlations. Cereb Cortex.

[27] Patel AX, Kundu P, Rubinov M (2014). A wavelet method for modeling and despiking motion artifacts from resting-state fMRI time series. Neuroimage.

[28] Smyser CD, Inder TE, Shimony JS (2010). Longitudinal analysis of neural network development in preterm infants. Cereb Cortex.

[29] Rabiner L, Juang B (1986). An introduction to hidden Markov models. IEEE ASSP Mag.

[30] Ray KL, McKay DR, Fox PM (2013). ICA model order selection of task coactivation networks. Front Neurosci.

[31] Vidaurre D, Llera A, Smith SM, Woolrich MW (2021). Behavioural relevance of spontaneous, transient brain network interactions in fMRI. Neuroimage.

[32] Quinn AJ, Vidaurre D, Abeysuriya R, Becker R, Nobre AC, Woolrich MW (2018). Task-evoked dynamic network analysis through hidden markov modeling. Front Neurosci.

[33] Shirer WR, Ryali S, Rykhlevskaia E, Menon V, Greicius MD (2012). Decoding subject-driven cognitive states with whole-brain connectivity patterns. Cereb Cortex.

[34] R Core Team R:A Language and Environment for Statistical Computing. R Foundation for Statistical Computing.

[35] Winkler AM, Ridgway GR, Webster MA, Smith SM, Nichols TE (2014). Permutation inference for the general linear model. Neuroimage.

[36] Bates D, Mächler M, Bolker B, Walker S (2015). Fitting linear mixed-effects models using lme4. J Stat Soft.

[37] Tsagris M, Preston S, Wood ATA (2016). Improved classification for compositional data using the α-transformation. J Classif.

[38] MacArthur RH (1957). On the relative abundance of bird species. Proc Natl Acad Sci USA.

[39] Moore KM, Nicholas J, Grossman M (2020). Age at symptom onset and death and disease duration in genetic frontotemporal dementia: an international retrospective cohort study. Lancet Neuro.

[40] Gregory S, Long JD, Klöppel S (2017). Operationalizing compensation over time in neurodegenerative disease. Brain.

[41] Klöppel S, Gregory S, Scheller E (2015). Compensation in Preclinical Huntington’s disease: evidence from the track-on HD study. EBioMedicine.

[42] Meeter LH, Kaat LD, Rohrer JD, van Swieten JC (2017). Imaging and fluid biomarkers in frontotemporal dementia. Nat Rev Neurol.

[43] Seeley WW, Crawford RK, Zhou J, Miller BL, Greicius MD (2009). Neurodegenerative diseases target large-scale human brain networks. Neuron.

[44] Zhou J, Greicius MD, Gennatas ED (2010). Divergent network connectivity changes in behavioural variant frontotemporal dementia and Alzheimer’s disease. Brain.

[45] Zhou J, Seeley WW (2014). Network dysfunction in Alzheimer’s disease and frontotemporal dementia: implications for psychiatry. Biol Psychiatry.

[46] Sridharan D, Levitin DJ, Menon V (2008). A critical role for the right fronto-insular cortex in switching between central-executive and defaultmode networks. Proc Natl Acad Sci.

[47] Bonnelle V, Ham TE, Leech R (2012). Salience network integrity predicts default mode network function after traumatic brain injury. Proc Natl Acad Sci.

[48] Lee SE, Khazenzon AM, Trujillo AJ (2014). Altered network connectivity in frontotemporal dementia with C9orf72 hexanucleotide repeat expansion. Brain.

[49] Bocchetta M, Gordon E, Cardoso MJ (2018). Thalamic atrophy in frontotemporal dementia—not just a C9orf72 problem. Neuroimage Clin.

[50] Lurie DJ, Kessler D, Bassett DS (2019). Questions and controversies in the study of time-varying functional connectivity in resting fMRI. Netw Neurosci.

