# Supplementary material for: Temporal dynamics predict symptom onset and cognitive decline in familial frontotemporal dementia
Source: Alzheimers Dement. 2022 Nov 15;19(5):1947–62. doi: 10.1002/alz.12824 (PMC7614527; doi:10.1002/alz.12824)
Supplement: Supplementary file 1 — SUPPORTING INFORMATION [file ALZ-19-1947-s001.docx]

Supplementary material

### Supplementary methods

**Image acquisition and preprocessing**

Participants from Cambridge underwent functional MRI imaging with at 3T using echo-planar imaging sensitive to the blood-oxygen-level-dependent signal (TR 2 secs, TE 30ms, whole brain acquisition, 3x3x3.75mm voxels, 155-305 volumes) with eyes open in a dark bore with a blank screen. High resolution T1-weighted Magnetization Prepared Rapid Gradient Echo (MPRAGE) structural images (TR 2-2.3s, TE 2.86-2.93ms, voxel size 1.1-1.25mm isotropic) were acquired during the same session for use in normalisation

For GENFI Echo-Planar Imaging and MPRAGE were acquired at each site at 3T or 1.5T where no 3T scanner was available, with corresponding imaging sequences across sites developed by the GENFI Imaging Core team. Echo-Planar Imaging was acquired with a median TR 2500ms, TE 30ms, median volume number 200 (range 122-305, upper and lower bound of interquartile range 200), in-plane resolution of 3x3mm and slice thickness of 3.5mm. T1 weighted MPRAGE structural images had a median isotropic resolution of 1.1mm, median TR 2s and median TE 2.9ms. Detailed acquisition times are provided in Supplementary Table 1.

We adapted the FSL preprocessing pipeline[1] used in previous work using hidden Markov modelling[2] on resting state fMRI data, with the addition of wavelet despiking[3] given higher in-scanner movement in participants with neurodegenerative diseases. For initial fMRI preprocessing the T1 structural images were cropped to remove non-brain tissue followed by brain extraction using FSL’s Brain Extraction Tool. We then used FSL’s FEAT with the following steps: motion correction using MCFLIRT; spatial smoothing using a Gaussian kernel of 5mm FWHW; grand-mean intensity normalisation of the 4D dataset by a single multiplicative factor; and 100Hz high-pass temporal filtering. Structured artefacts were removed using independent component analysis denoising using FSL’s MELODIC together with FIX. FIX was hand-trained using a set of 10 subjects from each disease group per cohort. Registration to high resolution structural and/or standard space images was carried out using FLIRT. Registration from high resolution structural to MNI space was then further refined using FNIRT nonlinear registration. We did not use global signal regression. Wavelet despiking was used for further removal of motion artefact.

**Comparing with canonical static resting state networks**

We compared mean activation maps with template maps of canonical resting state networks provided by Shirer et al [4]. We calculated the mean score within both a binarised template map and within a binarised inverse, with goodness of fit being the difference between the two. Higher scoring networks were taken to be matching resting state networks of positive areas of activation for that state, and strongly negative scores to be the corollaries of the negative state activations.

**Static resting state analysis**

To provide insight into the relationship between dynamic network changes and connectivity loss, we performed an additional analysis on the GENFI cohort to assess connectivity changes in resting state functional networks. We adopted the dual regression approach.[5] We performed a further independent component analysis using MELODIC with a lower model order of 15 to avoid fragmentation of large-scale networks. Components were matched to canonical resting state networks using the method outlined above. The first stage of dual regression involved regressing these group spatial maps into each participant’s fMRI 4-dimensional dataset to give participant specific timecourses. These timecourses were then used in a second regression with the component maps to obtain participant spatial maps per component. Assessment of differences in the spatial maps across groups used FSL’s randomise tool with 5000 permutations with age and sex included as covariates of no interest. We focused on four networks relevant to FTD: salience network; default mode network; left executive network; and right executive network. Resultant p-values were family-wise error (FWE) corrected for multiple comparisons across voxels. We furthermore calculated mean beta for each participant from individual component spatial maps from the second stage of dual regression using FSL’s *fslmeants* function, in order to compare between groups.

## Supplementary results

**Transition and persistence probabilities**

For the Cambridge dataset, a permutation test of persistence and transition probabilities found no group differences following correction for multiple comparisons. For GENFI, a permutation test of persistence and transition probabilities found a decreased transition probability in FTD from state 2 (salience) to state 3 (subcortical) (*t*(455)=4.5,  *P*=0.0002, Fig. 2C), and decreased persistence probability for state 3 (*t*(455)=3.7, *P*=0.007). We found an increased persistence probability in FTD for state 2 (*t*(455)=4.2, *P*=0.002), and increased transition probabilities from state 6 (default mode) to state 2 (*t*(455)=4.3, *P*=0.0008), from state 5 to state 4 (*t*(455)=3.2, *P*=0.046), from state 3 to both state 5 (*t*(455)=4.5, *P*=0.0002) and state 6 (*t*(455)=3.3, *P*=0.04) and from state 1 to state 6 (*t*(455)=4.5, *P*=0.0002).

**Principal component analysis for the Cambridge dataset**

For the Cambridge dataset two components were selected by MacArthur’s criterion, which explained 87% of the variance (Supplementary Fig. 3). Higher scores in the first component were associated with more time in states 2 and states 6, and less time in states 3 and states 5. Higher scores in the second component were associated with less time in states 1 and 4, and more time in state 6. Scores were significantly increased for the first component in FTD (F=4.1, *P*=0.046), with the group difference in second component scores not found to be significant (F=3.2, *P*=0.078).

**Variation by mutation and clinical phenotype**

In GENFI the behavioural variant frontotemporal dementia and primary progressive aphasia accounted for 83% of symptomatic patients, with the remaining patients split between twelve other diagnostic labels. Considering three groups (non-carriers, behavioural variant FTD and primary progressive aphasia) we found that fractional occupancy component scores were higher in both disease groups than in non-carriers (post-hoc Tukey PPA *t*=3.4, *P*=0.0019; bvFTD *t*=4.5 *P*<0.0001) but did not differ between the clinical phenotypes (*t*=0.68, *P*=0.78).

Assessing for variation in fractional occupancy component score by mutation type, we found no difference by mutation in symptomatic participants (*F*=1.27, *P*=0.29), and did not find that mutation type modified the relationship between symptomatic participants and non-carriers (*F*=1.3, *P*=0.27).

**Network dynamics and clinical correlates in symptomatic mutation carriers**

From Cambridge data, we found that scores for the first component in participants with FTD showed an uncorrected association with Addenbrooke’s Cognitive Examination-Revised (Supplementary Fig. 4A, Std Beta=-0.41, uncorrected *P*=0.039, FDR *P*=0.069) and Mini-Mental State Examination (Std Beta=-0.43, uncorrected *P*=0.035, FDR *P*=0.069). There were no significant associations with Frontal Assessment Battery score (Std Beta=-0.55, *P*=0.069) or Cambridge Behavioural Inventory-Revised (Std Beta=-0.01, *P*=0.96). No significant associations were observed with the second component.

For GENFI we found fractional occupancy component scores for symptomatic participants correlated with neuropsychological assessment (Supplementary Fig. 4B): digit symbol (Std Beta -0.21, *P*=0.019); trail making test B (Std Beta 0.22, *P*=0.019); backwards digit span (Std Beta -0.21, *P*=0.019); letter fluency (Std Beta -0.22, *P*=0.019); Boston naming test (Std Beta -0.19, *P*=0.034); and category fluency (Std Beta -0.21, *P*=0.019). No relationship was found with the Cambridge Behavioural Inventory-Revised (Std Beta=0.02, *P*=0.79) and MMSE (Std Beta -0.15, *P*=0.082)

Assessing for differences in slope between non-carriers and symptomatic patients using the interaction between component scores and group found significantly steeper slopes in trail making test B (interaction Std Beta -0.3, *P*=0.0004); MMSE (interaction Std Beta 0.27, *P*=0.007); and Boston naming (interaction Std Beta 0.25, *P*=0.011). The interaction was not significant for letter fluency (interaction Std Beta 0.14, *P*=0.14); category fluency (interaction Std Beta 0.17, *P*=0.059); backwards digit span (interaction Std Beta 0.13, *P*=0.18); digit symbol (interaction Std Beta 0.13, *P*=0.11); and Cambridge Behavioural Inventory-Revised (interaction Std Beta -0.01, *P*=0.86)

**Network dynamics and neuropsychological testing in presymptomatic mutation carriers**

We assessed whether fractional occupancy component scores correlated with preregistered neuropsychological assessments (trail making test B, digit symbol, backwards digit span) in presymptomatic mutation carriers. In presymptomatic mutation carriers component scores correlated with trail making test B (Std Beta=0.15, *P*=0.015) with no relationship found in non-carriers (Std Beta=0.01, *P*=0.92). Moreover, the relationship in presymptomatic mutation carriers was modified by age (interaction Std Beta=0.13, *P*=0.043). We found no relationship with component scores and either backwards digit span or digit symbol score.

**Salience network occupancy and longitudinal cognitive decline**

We assessed whether higher baseline saliency occupancy scores in symptomatic patients were associated with subsequent neurocognitive decline using pre-registered assessments (trail making test B, digit symbol, backwards digit span) and measures of global cognitive and behavioural decline (CBI-R, MMSE). Correcting for age at baseline scan, sex and site, baseline state 2 occupancy was related to the annual rate of clinical progression for MMSE (Fig. 3B, Std Beta=-0.4, *P*=0.003), backwards digit span (Std Beta=-0.34, *P*=0.008), digit symbol (Std Beta=-0.27, *P*=0.025) and trail making test B (Std Beta=0.4, *P*=0.025). No relationship was found with carer-rated severity using the CBI-R (Std Beta=0.09 *P*=0.45). We found a significant difference in slope between symptomatic mutation carriers and non-carriers for all measures except digit symbol and CBI-R (group x baseline state 2 interaction: MMSE Std Beta=-0.6, *P*=3x10^-9^; backwards digit span Std Beta=-0.27 *P*=0.0498; trail making test B Std Beta=0.57 *P*=6x10^-6^; CBI-R Std Beta=0.12 *P*=0.17; digit symbol Std Beta=-0.17 *P*=0.079).

We proceeded to investigate whether baseline salience network occupancy predicted cognitive and clinical decline in presymptomatic mutation carriers, hypothesising that the relationship between annualised rate of change in neurocognitive measure and baseline salience would depend on age as a marker of proximity to symptom onset.

We found that age significantly modified the relationship between annualised rate of clinical progression and baseline salience network occupancy for trail making test B (Interaction Std Beta=0.21 *P*=0.002), MMSE (Interaction Std Beta=-0.16 *P*=0.023) and the CBI-R (Interaction Std Beta=0.16 *P*=0.030). For these three measures, significant three-way interactions (group x age x state 2 occupancy) implied that baseline state 2 increased the rate of clinical deterioration in older presymptomatic mutation carriers, relative to non-carriers or younger carriers (Supplementary Table 4). We did not find any significant relationships with digit symbol or backwards digit span.

**Stability of results with differing independent component analysis model order**

We investigated whether the results of our hidden Markov modelling in the GENFI cohort were dependent on the initial model order chosen for the independent component analysis. We repeated our analysis with an independent component analysis where the number of dimensions were chose using MELODIC’s automated process.[6] 60 independent components were found, with 24 discarded as artefactual. We found that the newly identified states showed high cross-correlation with their most closely matching state in the primary analysis (spatial correlation of mean activation maps 0.77-0.85). We found that state occupancies were highly correlated with their corresponding state in the primary analysis (state occupancies R 0.84-0.94; component scores R=0.89).

**Impact of higher average motion participants on network dynamics**

To ensure that our results of the GENFI cohort were not distorted by participants who showed higher motion but were not excluded by our maximum-statistic based criteria, we repeated our analyses excluding 28 scans from 27 participants (10 non-carriers, 9 presymptomatic mutation carriers, 8 symptomatic carriers) who were above 1.2 standard deviations from the whole group mean for mean framewise displacement but included the primary analysis.

We found that component scores differed between symptomatic participants and non carriers (F=24.8 *P*=9x10^-7^). In symptomatic participants higher component scores were associated with digit symbol, trail making test B, backwards digit span and category fluency (Digit Symbol Std Beta -0.21 *P*=0.029; Trail making test B Std Beta 0.21 *P*=0.029; Digit span Std Beta -0.23 *P*=0.029*;* Category fluency Std Beta -0.21 *P*=0.029; MMSE Std Beta -0.18 *P*=0.062 ;CBI-R Std Beta 0.05 *P*=0.56; Letter Fluency -0.18 *P*=0.062, Boston naming Std Beta -0.17 *P*=0.062). Component scores were increased in converters at their latest presymptomatic scan (F=6.2 *P*=0.013). Baseline component scores in symptomatic participants were associated with longitudinal decline in MMSE (Std Beta -0.46, *P*=0.0006) and with uncorrected change in digit span (Std Beta -0.28 *P*=0.020, FDR *P*=0.051) and trail making test B (Std Beta 0.35, uncorrected *P*=0.035, FDR *P*=0.058). Baseline component scores were associated with cognitive decline in older presymptomatic mutation carriers for the MMSE and trial making test B (MMSE Interaction Std Beta -0.14 *P*=0.045; TMTB Std B 0.23 *P*=0.0004).

**Static resting state connectivity differences in the GENFI cohort**

We found greater within-network covariance in all four resting state networks of interest, with the most extensive changes found in the salience network and default mode network (Supplementary Fig. 5). Comparing scores derived from participant’s component spatial maps from the second stage of dual regression, we found that reduced connectivity scores in symptomatic participants for both the salience network (F=24.5 *P*=5x10^-11^; post-hoc Tukey non-carriers > symptomatic t=7.0 *P*<1x10^-7^ ; presymptomatic mutation carriers > symptomatic t=5.7 *P*=6x10^-8^) and the default mode network (F=11.3 *P*=1x10^-5^; post-hoc Tukey non-carriers> symptomatic t=4.1 *P*=0.0002 ; presymptomatic mutation carriers > symptomatic t=4.7 *P*=1x10^-5^).

**Supplementary Figure 1**

**
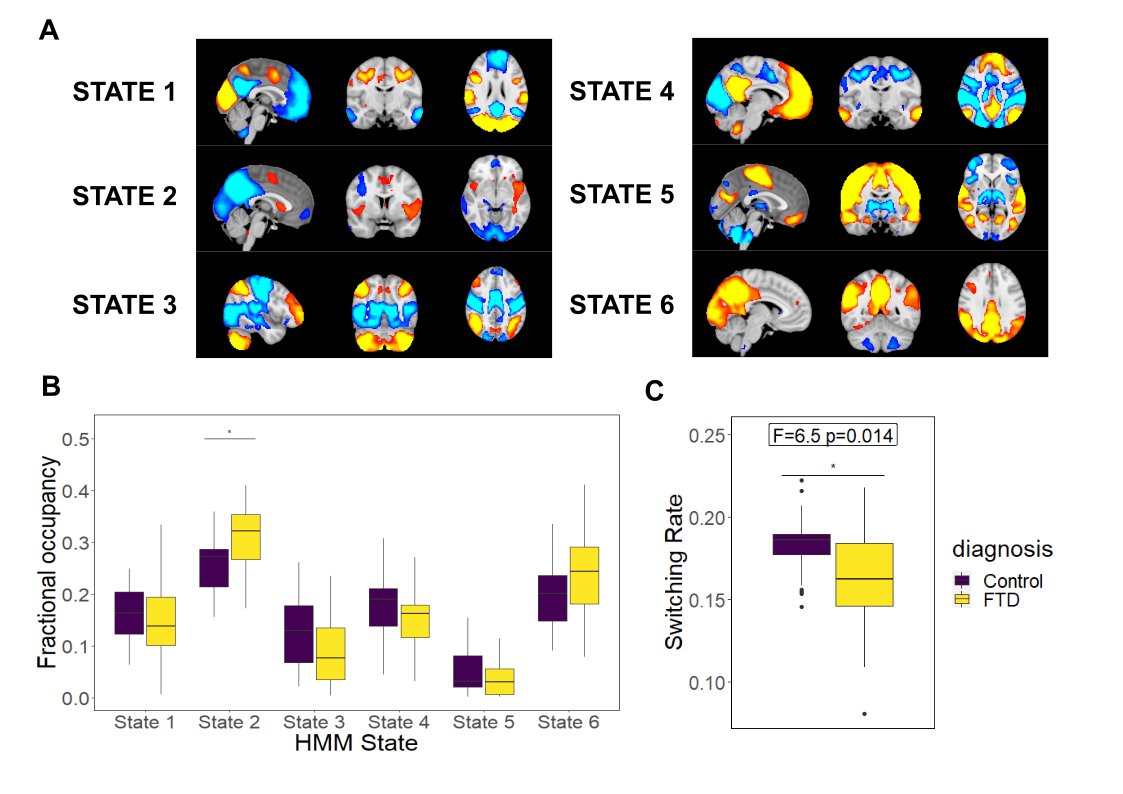
**

**Supplementary figure 1. Network dynamics in the Cambridge dataset. (A)** Mean activation maps for the six modelled states. **(B)** Fractional occupancy by state, with a post-correction increase in state 2 occupancy in frontotemporal dementia (FTD). **(C)** Switching rates are reduced in FTD in this cohort.

**Supplementary Figure 2**

**
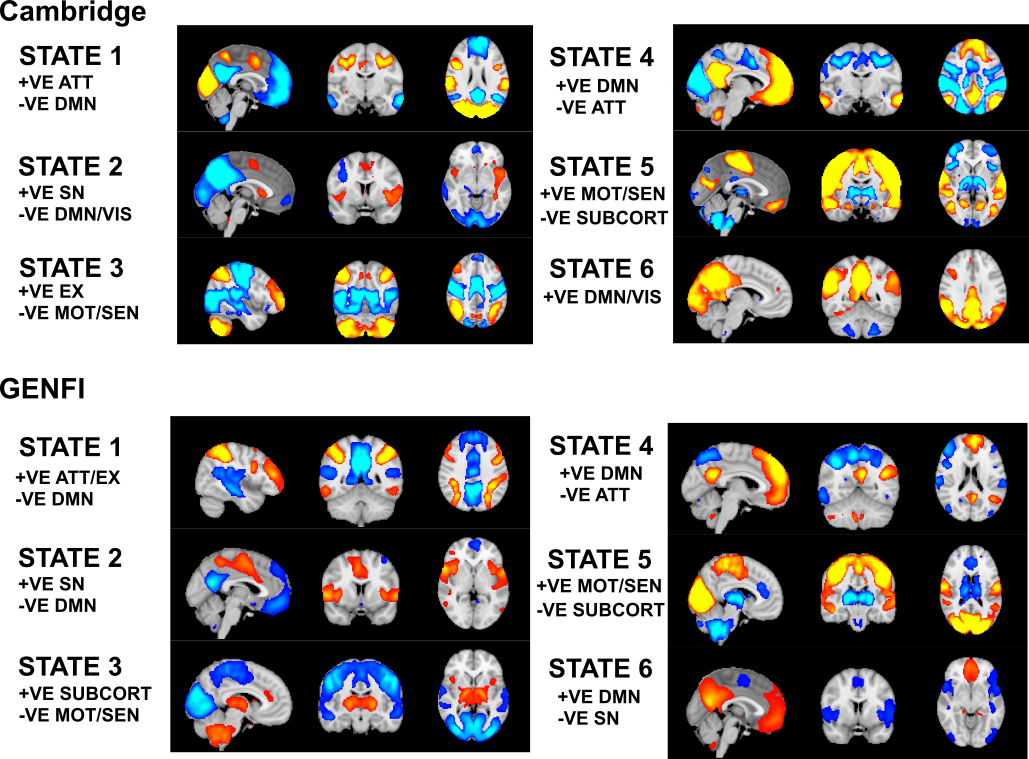
**

**Supplementary figure 2: Mean activation states for the two cohorts.** Mean activation maps for the six modelled states in each cohort, with reference to their closest canonical static functional network (DMN default mode network, SN salience network, EX Executive, ATT Attention, MOT motor, SEN sensory, VIS visual).

**Supplementary Figure 3**

**
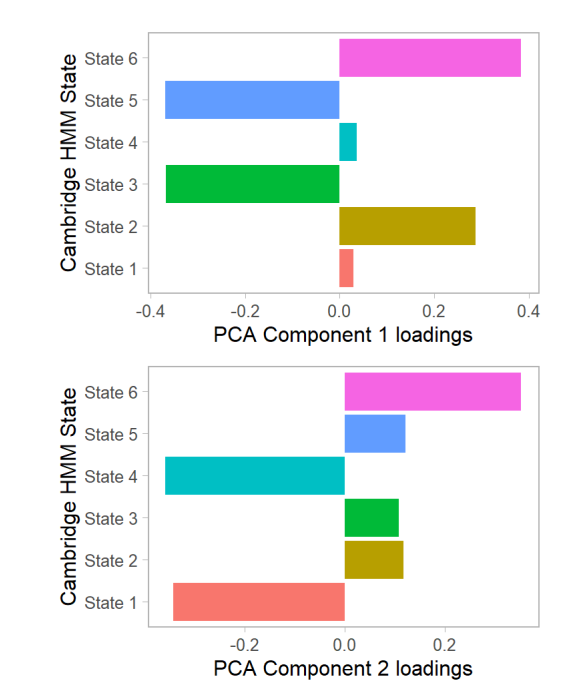
**

**Supplementary Figure 3: Principal component analysis loadings for state fractional occupancies for the Cambridge dataset** (PCA: Principal component analysis, HMM hidden Markov modelling)

**Supplementary Figure 4**

**
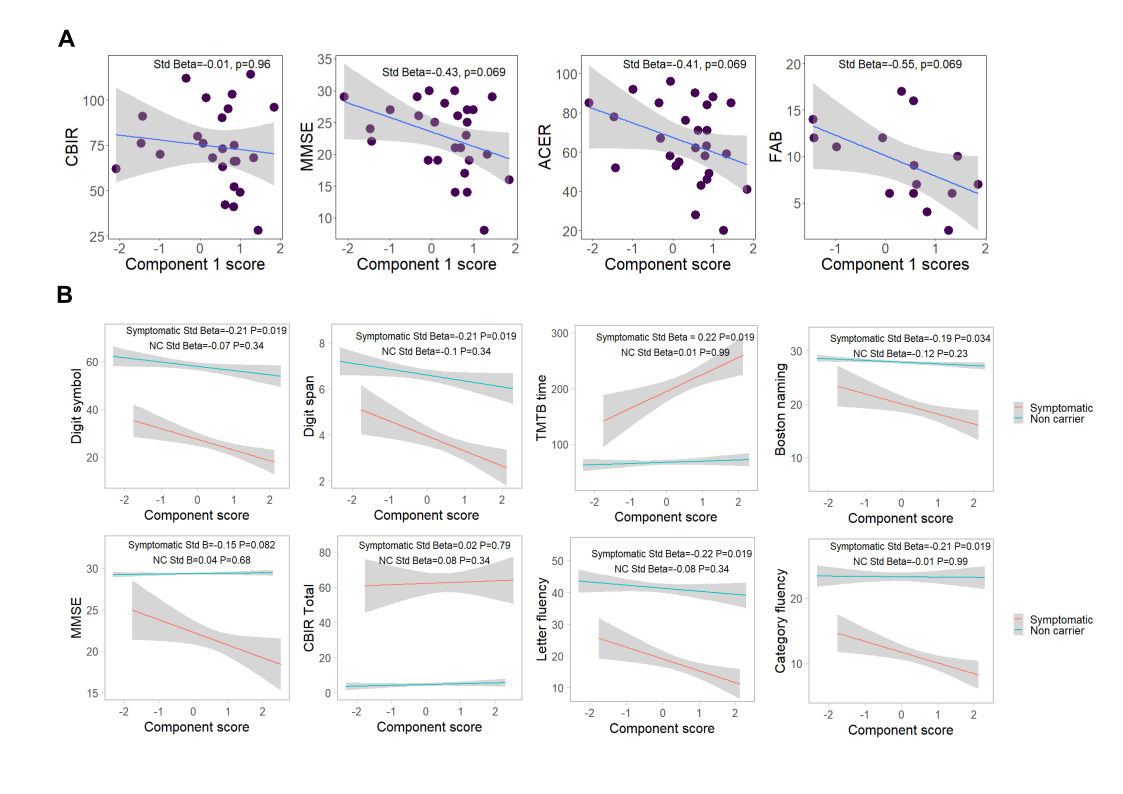
**

**Supplementary figure 4: Fractional occupancy component scores and neuropsychological assessments.** Component scores **(A)** showed uncorrected association with MMSE and Addenbrookes Cognitive Examination-Revised (ACE-R) in the Cambridge dataset and **(B)** with associations with neuropsychological measures in GENFI. Single subject data not plotted to protect genetic anonymity. Significant differences in slope were seen for TMTB, Boston naming, and MMSE. (CBIR Cambridge Behavioural Inventory-Revised, TMTB Trail Making Test B, MMSE Mini-Mental State Examination, FAB Frontal Assessment Battery)

**Supplementary Figure 5**

**
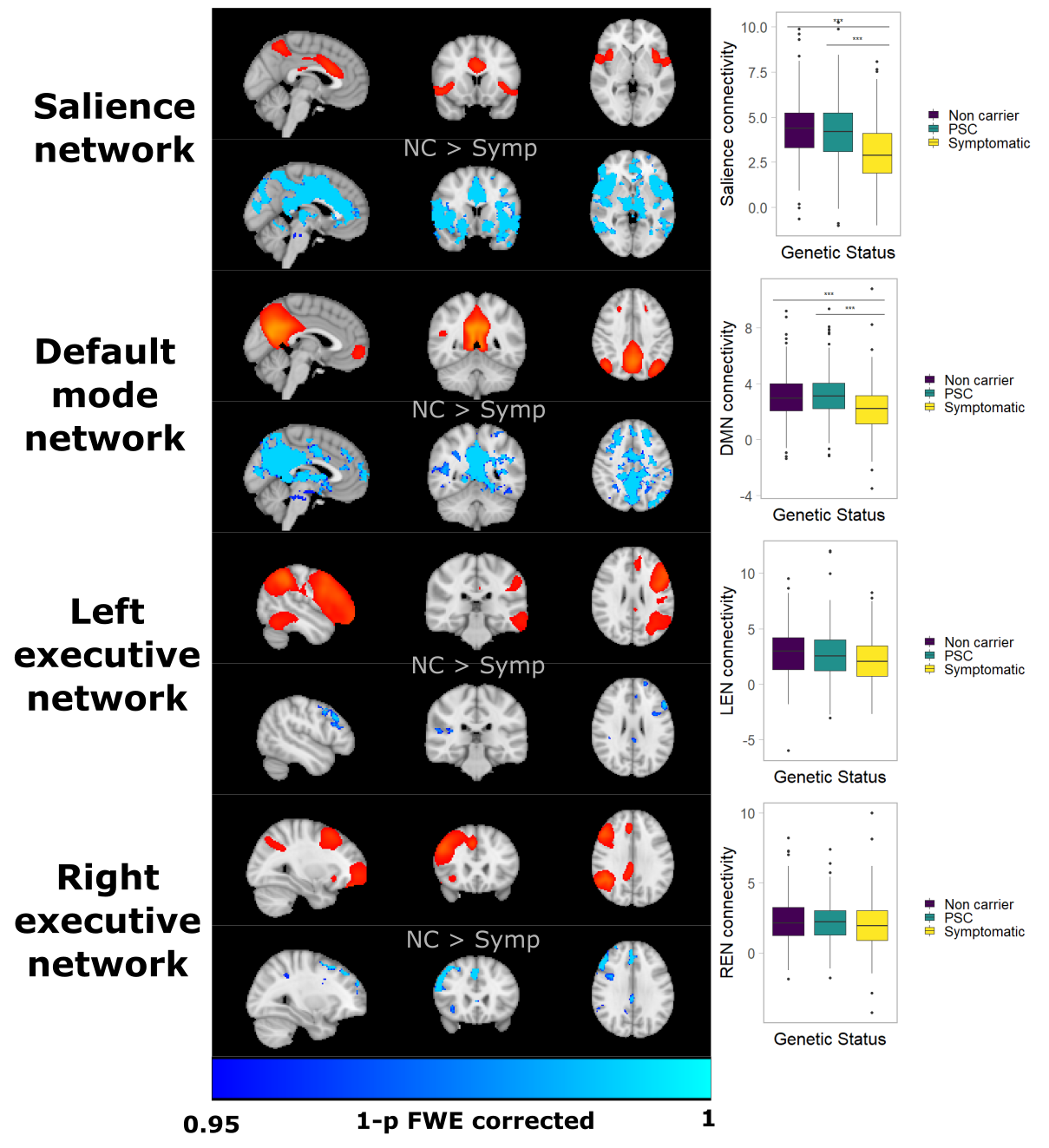
**

**Supplementary Figure 5. Dual regression analysis for the GENFI cohort**. Voxel-wise differences in network covariance (p<0.05 FWE corrected) in four selected resting state networks were observed. Comparing scores derived from participant’s individual component spatial maps, we found reduced scores in symptomatic participants in the salience network and default mode network (NC: Non-carrier, PSC: Pre-symptomatic mutation carrier, Symp: Symptomatic mutation carrier)**Supplementary Table 1: MRI acquisition parameters of all included GENFI scans**

| **Scans**  **(n)** | **Scanner** | **TR (s)** | **TE** | **Volumes** | **Volume slices** | **Slice thickness** | **Pixel spacing** | **FOV** | **Field strength** |
| --- | --- | --- | --- | --- | --- | --- | --- | --- | --- |
| 88 | Philips Achieva | 2.2 | 30 | 140 | 36 | 3.3 | 2.65\2.65 | 80*80 | 3 |
| 197 | Philips Achieva | 2.2 | 30 | 200 | 38 | 2.72 | 2.75\2.75 | 80*80 | 3 |
| 73 | Philips Achieva | 2.5 | 30 | 200 | 42 | 3.5 | 3\3 | 64*64 | 3 |
| 58 | Philips Achieva | 2.5 | 30 | 200 | 42 | 3.5 | 3\3 | 64*64 | 3 |
| 15 | Philips Achieva | 2.2 | 30 | 140 | 33 | 3.5 | 2.3\2.3 | 96*96 | 3 |
| 2 | Philips Achieva | 2.2 | 30 | 140 | 36 | 3.3 | 2.5\2.5 | 96*96 | 3 |
| 1 | Philips Achieva | 2.2 | 30 | 140 | 42 | 3.3 | 2.5\2.5 | 96*96 | 3 |
| 9 | Siemens Aera | 3 | 30 | 200 | 29 | 3 | 3.4\3.4 | 64*64 | 1.5 |
| 3 | Siemens Allegra | 2.2 | 30 | 140 | 36 | 3.4 | 3.4\3.4 | 64*64 | 3 |
| 36 | Siemens Avanto | 2.2 | 30 | 200 | 29 | 3.5 | 3.5\3.5 | 64*64 | 1.5 |
| 1 | Siemens Avanto | 2.2 | 30 | 200 | 29 | 3.5 | 3.7\3.7 | 64*64 | 1.5 |
| 47 | GE Discovery | 2.2 | 30 | 140 | 39 | 3.3 | 3.4\3.4 | 64*64 | 3 |
| 329 | Siemens Prisma | 2.5 | 30 | 200 | 42 | 3.5 | 3\3 | 64*64 | 3 |
| 38 | Siemens Prisma | 2.5 | 30 | 200 | 42 | 3.5 | 3\3 | 64*58 | 3 |
| 14 | Siemens Prisma | 2.2 | 30 | 140 | 36 | 3.3 | 3.3\3.3 | 64*58 | 3 |
| 1 | Siemens Prisma | 2.5 | 30 | 200 | 42 | 3.5 | 3.3\3.3 | 64*64 | 3 |
| 1 | GE Signa | 2.5 | 30 | 200 | 36 | 3.5 | 3\3 | 64*64 | 3 |
| 8 | GE Signa | 3 | 30 | 200 | 40 | 3.5 | 3.4\3.4 | 64*64 | 1.5 |
| 1 | GE Signa | 2.5 | 30 | 200 | 45 | 3.5 | 3\3 | 64*64 | 3 |
| 1 | GE Signa | 3 | 30 | 200 | 39 | 3.5 | 3.4\3.4 | 64*64 | 1.5 |
| 123 | Simens Skyra | 2.5 | 30 | 200 | 42 | 3.5 | 3\3 | 64*64 | 3 |
| 86 | Simens Skyra | 2.5 | 30 | 200 | 42 | 3.5 | 3\3 | 64*58 | 3 |
| 16 | Simens Skyra | 2.25 | 30 | 140 | 36 | 3.3 | 3.3\3.3 | 64*58 | 3 |
| 2 | Simens Skyra | 2.5 | 30 | 200 | 45 | 3.5 | 3\3 | 64*58 | 3 |
| 2 | Simens Skyra | 2.5 | 30 | 200 | 42 | 3.5 | 3\3 | 64*60 | 3 |
| 1 | Simens Skyra | 2.5 | 30 | 200 | 46 | 3.5 | 3\3 | 64*60 | 3 |
| 3 | Simens Skyra | 2.5 | 30 | 200 | 42 | 3.5 | 3.4\3.4 | 64*64 | 3 |
| 2 | Simens Skyra | 2.5 | 30 | 200 | 42 | 3.5 | 3.4\3.4 | 64*58 | 3 |
| 2 | Simens Skyra | 2.5 | 30 | 200 | 42 | 3.5 | 3.1\3.1 | 64*58 | 3 |
| 3 | Siemens Triotrim | 2 | 30 | 300 | 32 | 3 | 3\3 | 64*64 | 3 |
| 137 | Siemens Triotrim | 2.2 | 30 | 140 | 42 | 3.3 | 3.3\3.3 | 64*58 | 3 |
| 254 | Siemens Triotrim | 2.5 | 30 | 200 | 42 | 3.4 | 3\3 | 64*64 | 3 |
| 2 | Siemens Triotrim | 2.5 | 30 | 200 | 46 | 3.4 | 3\3 | 64*64 | 3 |
| 1 | Siemens Triotrim | 2.2 | 30 | 140 | 36 | 3.3 | 3.3\3.3 | 64*60 | 3 |

TR=Repetition time, TE=Echo time, FOV=Field-of-view

**Supplementary Table 2: Linear v Quadratic model comparison for age against state occupancy and component scores for all carriers, presymptomatic carriers and non-carriers.**

| **State** | **All carriers** | | **Presymptomatic carriers** | | **Non carriers** | |
| --- | --- | --- | --- | --- | --- | --- |
|  | **ChiSq** | **P** | **ChiSq** | **P** | **ChiSq** | **P** |
| **State 1** | 0.03 | 0.96 | 0.15 | 0.70 | 0.14 | 0.91 |
| **State 2** | 8.7 | 0.020 | 7.2 | 0.043 | 0.03 | 0.91 |
| **State 3** | 2.5 | 0.35 | 1.3 | 0.70 | 0.29 | 0.91 |
| **State 4** | 0.0 | 0.96 | 0.24 | 0.70 | 1.6 | 0.62 |
| **State 5** | 1.7 | 0.39 | 0.74 | 0.70 | 0.01 | 0.91 |
| **State 6** | 0.01 | 0.96 | 0.23 | 0.70 | 1.6 | 0.62 |
| **PCA** | 3.5 | 0.061 | 3.2 | 0.073 | 0.05 | 0.83 |

PCA: Principal component analysis component. State P values corrected for false discovery rate across states

**Supplementary table 3. Annualised rates of change in clinical assessments**

| **Neurocognitive/clinical assessment** | **Annualised rate of change** | | |
| --- | --- | --- | --- |
| Group | Symptomatic | Presymptomatic mutation carriers | Non-carriers |
| TMTB | 20 (16) | 0.65 (5.6) | 0.33 (4.7) |
| Digit span | -0.05 (0.06) | 0.02 (0.06) | 0.01 (0.05) |
| Digit symbol | -1.1 (0.79) | 0.54 (0.79) | 0.61 (0.69) |
| MMSE | -2.1 (1.9) | -0.04 (0.22) | -0.02 (0.18) |
| CBI-revised | 5.9 (3.7) | 0.68 (1.5) | 0.52 (0.89) |

Scores are mean (SD). (TMTB Trail Making Test B, CBIR Cambridge Behavioural Inventory-Revised, MMSE Mini-Mental State Examination)

**Supplementary Table 4 Two step prediction models for presymptomatic mutation carriers v baseline state 2 (salience state) occupancy**

| **Model** | **Slope ~ state 2 + cov** | | | **Slope ~ state 2*age + cov** | | | **Slope ~ state2*age*group + cov** | | |
| --- | --- | --- | --- | --- | --- | --- | --- | --- | --- |
|  | *Std Beta* | *t* | *P* | *Std Beta* | *t* | *P* | *Std Beta* | *t* | *P* |
| **TMTB** | 0.12 | 1.9 | 0.26 | 0.21 | 3.6 | 0.002 | 0.38 | 4.4 | 0.0008 |
| **Digit span** | 0.01 | 0.16 | 0.87 | 0.07 | 1.2 | 0.30 | 0.1 | 1.1 | 0.27 |
| **Digit symbol** | -0.05 | -0.97 | 0.55 | 0 | 0.08 | 0.93 | -0.09 | -1.2 | 0.27 |
| **MMSE** | -0.07 | -1.2 | 0.55 | -0.16 | -2.6 | 0.024 | -0.24 | -2.7 | 0.022 |
| **CBIR** | 0.05 | 0.8 | 0.55 | 0.16 | 2.4 | 0.030 | 0.22 | 2.2 | 0.032 |

(TMTB Trail Making Test B, CBIR Cambridge Behavioural Inventory-Revised, MMSE Mini-Mental State Examination)

### Supplementary references

[1] Smith SM, Beckmann CF, Andersson J, Auerbach EJ, Bijsterbosch J, Douaud G, et al. Resting-state fMRI in the Human Connectome Project. NeuroImage 2013;80:144–68. https://doi.org/10.1016/j.neuroimage.2013.05.039.

[2] Vidaurre D, Smith SM, Woolrich MW. Brain network dynamics are hierarchically organized in time. Proceedings of the National Academy of Sciences 2017;114:12827. https://doi.org/10.1073/pnas.1705120114.

[3] Patel AX, Kundu P, Rubinov M, Jones PS, Vértes PE, Ersche KD, et al. A wavelet method for modeling and despiking motion artifacts from resting-state fMRI time series. NeuroImage 2014;95:287–304. https://doi.org/10.1016/j.neuroimage.2014.03.012.

[4] Shirer WR, Ryali S, Rykhlevskaia E, Menon V, Greicius MD. Decoding Subject-Driven Cognitive States with Whole-Brain Connectivity Patterns. Cerebral Cortex 2012;22:158–65. https://doi.org/10.1093/cercor/bhr099.

[5] Filippini N, MacIntosh BJ, Hough MG, Goodwin GM, Frisoni GB, Smith SM, et al. Distinct patterns of brain activity in young carriers of the *APOE* -ε4 allele. Proc Natl Acad Sci USA 2009;106:7209–14. https://doi.org/10.1073/pnas.0811879106.

[6] Beckmann CF, Smith SM. Probabilistic Independent Component Analysis for Functional Magnetic Resonance Imaging. IEEE Trans Med Imaging 2004;23:137–52. https://doi.org/10.1109/TMI.2003.822821.
