# Supplementary material for: Temporal dynamics predict symptom onset and cognitive decline in familial frontotemporal dementia
Source: Alzheimers Dement. Author manuscript; Available in PMC 2023 May 12. (PMC7614527; doi:10.1002/alz.12824)
Supplement: Supplementary file 1 [file EMS172241-supplement-Appendix.pdf]

APPENDIX

GENFI consortium authors

Aitana Sogorb Esteve (Department of Neurodegenerative Disease, Dementia Research Centre, UCL Queen Square Institute of Neurology, London, UK; UK Dementia Research Institute at University College London, UCL Queen Square Institute of Neurology, London, UK)
Annabel Nelson (Department of Neurodegenerative Disease, Dementia Research Centre, UCL Queen Square Institute of Neurology, London, UK)
Arabella Bouzigues (Department of Neurodegenerative Disease, Dementia Research Centre, UCL Queen Square Institute of Neurology, London, UK)
Carolin Heller (Department of Neurodegenerative Disease, Dementia Research Centre, UCL Queen Square Institute of Neurology, London, UK)
Caroline V Greaves (Department of Neurodegenerative Disease, Dementia Research Centre, UCL Queen Square Institute of Neurology, London, UK)
David Cash (Department of Neurodegenerative Disease, Dementia Research Centre, UCL Queen Square Institute of Neurology, London, UK)
David L Thomas (Neuroimaging Analysis Centre, Department of Brain Repair and Rehabilitation, UCL Institute of Neurology, Queen Square, London, UK)
Emily Todd (Department of Neurodegenerative Disease, Dementia Research Centre, UCL Queen Square Institute of Neurology, London, UK)
Hanya Benotmane (UK Dementia Research Institute at University College London, UCL Queen Square Institute of Neurology, London, UK)
Henrik Zetterberg (UK Dementia Research Institute at University College London, UCL Queen Square Institute of Neurology, London, UK Department of Psychiatry and Neurochemistry,; the Sahlgrenska Academy at the University of Gothenburg, Mölndal, Sweden)
Imogen J Swift (Department of Neurodegenerative Disease, Dementia Research Centre, UCL Queen Square Institute of Neurology, London, UK; UK Dementia Research Institute at University College London, UCL Queen Square Institute of Neurology, London, UK)
Jennifer Nicholas (Department of Medical Statistics, London School of Hygiene and Tropical Medicine, London, UK)
Kiran Samra (Department of Neurodegenerative Disease, Dementia Research Centre, UCL Queen Square Institute of Neurology, London, UK)
Lucy L Russell (Department of Neurodegenerative Disease, Dementia Research Centre, UCL Queen Square Institute of Neurology, London, UK)
Martina Bocchetta (Department of Neurodegenerative Disease, Dementia Research Centre, UCL Queen Square Institute of Neurology, London, UK)
Rachelle Shafei (Department of Neurodegenerative Disease, Dementia Research Centre, UCL Queen Square Institute of Neurology, London, UK)
Rhian S Convery (Department of Neurodegenerative Disease, Dementia Research Centre, UCL Queen Square Institute of Neurology, London, UK)
Carolyn Timberlake (Department of Clinical Neurosciences, University of Cambridge, Cambridge, UK)
Thomas Cope (Department of Clinical Neuroscience, University of Cambridge, Cambridge, UK)
Timothy Rittman (Department of Clinical Neurosciences, University of Cambridge, Cambridge, UK)
Alberto Benussi (Centre for Neurodegenerative Disorders, Department of Clinical and Experimental Sciences, University of Brescia, Brescia, Italy)
Enrico Premi (Stroke Unit, ASST Brescia Hospital, Brescia, Italy)
Roberto Gasparotti (Neuroradiology Unit, University of Brescia, Brescia, Italy)
Silvana Archetti (Biotechnology Laboratory, Department of Diagnostics, ASST Brescia Hospital, Brescia, Italy)
Stefano Gazzina (Neurology, ASST Brescia Hospital, Brescia, Italy)
Valentina Cantoni (Centre for Neurodegenerative Disorders, Department of Clinical and Experimental Sciences, University of Brescia, Brescia, Italy)
Andrea Arighi (Fondazione IRCCS Ca' Granda Ospedale Maggiore Policlinico, Neurodegenerative Diseases Unit, Milan, Italy; University of Milan, Centro Dino Ferrari, Milan, Italy)
Chiara Fenoglio (Fondazione IRCCS Ca' Granda Ospedale Maggiore Policlinico, Neurodegenerative Diseases Unit, Milan, Italy; University of Milan, Centro Dino Ferrari, Milan, Italy)
Elio Scarpini (Fondazione IRCCS Ca' Granda Ospedale Maggiore Policlinico, Neurodegenerative Diseases Unit, Milan, Italy; University of Milan, Centro Dino Ferrari, Milan, Italy)
Giorgio Fumagalli (Fondazione IRCCS Ca' Granda Ospedale Maggiore Policlinico, Neurodegenerative Diseases Unit, Milan, Italy; University of Milan, Centro Dino Ferrari, Milan, Italy)
Vittoria Borracci
Giacomina Rossi (Fondazione IRCCS Istituto Neurologico Carlo Besta, Milano, Italy)
Giorgio Giaccone (Fondazione IRCCS Istituto Neurologico Carlo Besta, Milano, Italy)
Giuseppe Di Fede (Fondazione IRCCS Istituto Neurologico Carlo Besta, Milano, Italy)

15525279, 0, Downloaded from https://alz-journals.onlinelibrary.wiley.com/doi/10.1002/alz.12824 by Test, Wiley Online Library on [14/03/2023]. See the Terms and Conditions (https://onlinelibrary.wiley.com/terms-and-conditions) on Wiley Online Library for rules of use; OA articles are governed by the applicable Creative Commons License

Paola Caroppo (Fondazione IRCCS Istituto Neurologico Carlo Besta, Milano, Italy)
Pietro Tiraboschi (Fondazione IRCCS Istituto Neurologico Carlo Besta, Milano, Italy)
Sara Prioni (Fondazione IRCCS Istituto Neurologico Carlo Besta, Milano, Italy)
Veronica Redaelli (Fondazione IRCCS Istituto Neurologico Carlo Besta, Milano, Italy)
David Tang-Wai (The University Health Network, Krembil Research Institute, Toronto, Canada)
Ekaterina Rogaeva (Tanz Centre for Research in Neurodegenerative Diseases, University of Toronto, Toronto, Canada)
Miguel Castelo-Branco (Faculty of Medicine, University of Coimbra, Coimbra, Portugal)
Morris Freedman (Baycrest Health Sciences, Rotman Research Institute, University of Toronto, Toronto, Canada)
Ron Keren (The University Health Network, Toronto Rehabilitation Institute, Toronto, Canada)
Sandra Black (Sunnybrook Health Sciences Centre, Sunnybrook Research Institute, University of Toronto, Toronto, Canada)
Sara Mitchell (Sunnybrook Health Sciences Centre, Sunnybrook Research Institute, University of Toronto, Toronto, Canada)
Christen Shoesmith (Department of Clinical Neurological Sciences, University of Western Ontario, London, Ontario, Canada)
Robart Bartha (Department of Medical Biophysics, The University of Western Ontario, London, Ontario, Canada; Centre for Functional and Metabolic Mapping, Robarts Research Institute, The University of Western Ontario, London, Ontario, Canada)
Rosa Rademakers (Center for Molecular Neurology, University of Antwerp)
Jackie Poos (Department of Neurology, Erasmus Medical Center, Rotterdam, Netherlands)
Janne M. Papma (Department of Neurology, Erasmus Medical Center, Rotterdam, Netherlands)
Lucia Giannini (Department of Neurology, Erasmus Medical Center, Rotterdam, Netherlands)
Rick van Minkelen (Department of Clinical Genetics, Erasmus Medical Center, Rotterdam, Netherlands)
Yolande Pijnenburg (Amsterdam University Medical Centre, Amsterdam VUmc, Amsterdam, Netherlands)
Camilla Ferrari (Department of Neuroscience, Psychology, Drug Research and Child Health, University of Florence, Florence, Italy)
Cristina Polito (Department of Biomedical, Experimental and Clinical Sciences "Mario Serio", Nuclear Medicine Unit, University of Florence, Florence, Italy)
Gemma Lombardi (Department of Neuroscience, Psychology, Drug Research and Child Health, University of Florence, Florence, Italy)
Valentina Bessi (Department of Neuroscience, Psychology, Drug Research and Child Health, University of Florence, Florence, Italy)
Michele Veldsman (Nuffield Department of Clinical Neurosciences, Medical Sciences Division, University of Oxford, Oxford, UK)
Christin Andersson (Department of Clinical Neuroscience, Karolinska Institutet, Stockholm, Sweden)
Hakan Thonberg (Center for Alzheimer Research, Division of Neurogeriatrics, Karolinska Institutet, Stockholm, Sweden)
Linn Öijerstedt (Center for Alzheimer Research, Division of Neurogeriatrics, Department of Neurobiology, Care Sciences and Society, Bioclinicum, Karolinska Institutet, Solna, Sweden; Unit for Hereditary Dementias, Theme Aging, Karolinska University Hospital, Solna, Sweden)
Vesna Jelic (Division of Clinical Geriatrics, Karolinska Institutet, Stockholm, Sweden)
Paul Thompson (Division of Neuroscience and Experimental Psychology, Wolfson Molecular Imaging Centre, University of Manchester, Manchester, UK)
Tobias Langheinrich (Division of Neuroscience and Experimental Psychology, Wolfson Molecular Imaging Centre, University of Manchester, Manchester, UK Manchester Centre for Clinical Neurosciences, Department of Neurology, Salford Royal NHS Foundation Trust, Manchester, UK)
Albert Lladó (Alzheimer's disease and Other Cognitive Disorders Unit, Neurology Service, Hospital Clínic, Barcelona, Spain)
Anna Antonell (Alzheimer's disease and Other Cognitive Disorders Unit, Neurology Service, Hospital Clínic, Barcelona, Spain)
Jaume Olives (Alzheimer's disease and Other Cognitive Disorders Unit, Neurology Service, Hospital Clínic, Barcelona, Spain)
Mircea Balasa (Alzheimer's disease and Other Cognitive Disorders Unit, Neurology Service, Hospital Clínic, Barcelona, Spain)
Nuria Bargalló (Imaging Diagnostic Center, Hospital Clínic, Barcelona, Spain)
Sergi Borrego-Ecija (Alzheimer's disease and Other Cognitive Disorders Unit, Neurology Service, Hospital Clínic, Barcelona, Spain)
Ana Verdelho (Department of Neurosciences and Mental Health, Centro Hospitalar Lisboa Norte - Hospital de Santa Maria & Faculty of Medicine, University of Lisbon, Lisbon, Portugal)
Carolina Maruta (Laboratory of Language Research, Centro de Estudos Egas Moniz, Faculty of Medicine, University of Lisbon, Lisbon, Portugal)
Catarina B. Ferreira (Laboratory of Neurosciences, Faculty of Medicine, University of Lisbon, Lisbon, Portugal)

15525279, 0, Downloaded from https://alz-journals.onlinelibrary.wiley.com/doi/10.1002/alz.12824 by Test, Wiley Online Library on [14/03/2023]. See the Terms and Conditions (https://onlinelibrary.wiley.com/terms-and-conditions) on Wiley Online Library for rules of use; OA articles are governed by the applicable Creative Commons License

Gabriel Miltenberger (Faculty of Medicine, University of Lisbon, Lisbon, Portugal)
Frederico Simões do Couto (Faculdade de Medicina, Universidade Católica Portuguesa)
Alazne Gabilondo (Cognitive Disorders Unit, Department of Neurology, Donostia University Hospital, San Sebastian, Gipuzkoa, Spain; Neuroscience Area, Biodonostia Health Research Institute, San Sebastian, Gipuzkoa, Spain)
Ana Gorostidi (Neuroscience Area, Biodonostia Health Research Institute, San Sebastian, Gipuzkoa, Spain)
Jorge Villanua (OSATEK, University of Donostia, San Sebastian, Gipuzkoa, Spain)
Marta Cañada (CITA Alzheimer, San Sebastian, Gipuzkoa, Spain)
Mikel Tainta (Neuroscience Area, Biodonostia Health Research Institute, San Sebastian, Gipuzkoa, Spain)
Miren Zulaica (Neuroscience Area, Biodonostia Health Research Institute, San Sebastian, Gipuzkoa, Spain)
Myriam Barandiaran (Cognitive Disorders Unit, Department of Neurology, Donostia University Hospital, San Sebastian, Gipuzkoa, Spain; Neuroscience Area, Biodonostia Health Research Institute, San Sebastian, Gipuzkoa, Spain)
Patricia Alves (Neuroscience Area, Biodonostia Health Research Institute, San Sebastian, Gipuzkoa, Spain; Department of Educational Psychology and Psychobiology, Faculty of Education, International University of La Rioja, Logroño, Spain)
Benjamin Bender (Department of Diagnostic and Interventional Neuroradiology, University of Tübingen, Tübingen, Germany)
Carlo Wilke (Department of Neurodegenerative Diseases, Hertie-Institute for Clinical Brain Research and Center of Neurology, University of Tübingen, Tübingen, Germany; Center for Neurodegenerative Diseases (DZNE), Tübingen, Germany)
Lisa Graf (Department of Neurodegenerative Diseases, Hertie-Institute for Clinical Brain Research and Center of Neurology, University of Tübingen, Tübingen, Germany)
Annick Vogels (Department of Human Genetics, KU Leuven, Leuven, Belgium)
Mathieu Vandenbulcke (Geriatric Psychiatry Service, University Hospitals Leuven, Belgium; Neuropsychiatry, Department of Neurosciences, KU Leuven, Leuven, Belgium)
Philip Van Damme (Neurology Service, University Hospitals Leuven, Belgium; Laboratory for Neurobiology, VIB-KU Leuven Centre for Brain Research, Leuven, Belgium)
Rose Bruffaerts (Department of Biomedical Sciences, University of Antwerp, Antwerp, Belgium; Biomedical Research Institute, Hasselt University, 3500 Hasselt, Belgium)
Koen Poesen (Laboratory for Molecular Neurobiomarker Research, KU Leuven, Leuven, Belgium)
Pedro Rosa-Neto (Translational Neuroimaging Laboratory, McGill Centre for Studies in Aging, McGill University, Montreal, Québec, Canada)
Serge Gauthier (Alzheimer Disease Research Unit, McGill Centre for Studies in Aging, Department of Neurology & Neurosurgery, McGill University, Montreal, Québec, Canada)
Agnès Camuzat (Sorbonne Université, Paris Brain Institute – Institut du Cerveau – ICM, Inserm U1127, CNRS UMR 7225, AP-HP - Hôpital Pitié-Salpêtrière, Paris, France)
Alexis Brice (Sorbonne Université, Paris Brain Institute – Institut du Cerveau – ICM, Inserm U1127, CNRS UMR 7225, AP-HP - Hôpital Pitié-Salpêtrière, Paris, France; Reference Network for Rare Neurological Diseases (ERN-RND))
Anne Bertrand (Sorbonne Université, Paris Brain Institute – Institut du Cerveau – ICM, Inserm U1127, CNRS UMR 7225, AP-HP - Hôpital Pitié-Salpêtrière, Paris, FranceInria, Aramis project-team, F-75013, Paris, France; Centre pour l'Acquisition et le Traitement des Images, Institut du Cerveau et la Moelle, Paris, France)
Aurélie Funkiewiez (Centre de référence des démences rares ou précoces, IM2A, Département de Neurologie, AP-HP - Hôpital Pitié-Salpêtrière, Paris, France; Sorbonne Université, Paris Brain Institute – Institut du Cerveau – ICM, Inserm U1127, CNRS UMR 7225, AP-HP - Hôpital Pitié-Salpêtrière, Paris, France)
Daisy Rinaldi (Centre de référence des démences rares ou précoces, IM2A, Département de Neurologie, AP-HP - Hôpital Pitié-Salpêtrière, Paris, France; Sorbonne Université, Paris Brain Institute – Institut du Cerveau – ICM, Inserm U1127, CNRS UMR 7225, AP-HP - Hôpital Pitié-Salpêtrière, Paris, France; Département de Neurologie, AP-HP - Hôpital Pitié-Salpêtrière, Paris, France)
Olivier Colliot (Sorbonne Université, Paris Brain Institute – Institut du Cerveau – ICM, Inserm U1127, CNRS UMR 7225, AP-HP - Hôpital Pitié-Salpêtrière, Paris, France; Inria, Aramis project-team, F-75013, Paris, France; Centre pour l'Acquisition et le Traitement des Images, Institut du Cerveau et la Moelle, Paris, France)
Sabrina Sayah (Sorbonne Université, Paris Brain Institute – Institut du Cerveau – ICM, Inserm U1127, CNRS UMR 7225, AP-HP - Hôpital Pitié-Salpêtrière, Paris, France)
Catharina Prix (Neurologische Klinik, Ludwig-Maximilians-Universität München, Munich, Germany)

15525279, 0, Downloaded from https://alz-journals.onlinelibrary.wiley.com/doi/10.1002/alz.12824 by Test, Wiley Online Library on [14/03/2023]. See the Terms and Conditions (https://onlinelibrary.wiley.com/terms-and-conditions) on Wiley Online Library for rules of use; OA articles are governed by the applicable Creative Commons License

Elisabeth Wlasich (Neurologische Klinik, Ludwig-Maximilians-Universität München, Munich, Germany)
Olivia Wagemann
Sandra Loosli (Neurologische Klinik, Ludwig-Maximilians-Universität München, Munich, Germany)
Sonja Schönecker (Neurologische Klinik, Ludwig-Maximilians-Universität München, Munich, Germany)
Tobias Hoegen (Neurologische Klinik, Ludwig-Maximilians-Universität München, Munich, Germany)
Jolina Lombardi (Department of Neurology, University of Ulm, Ulm)
Sarah Anderl-Straub (Department of Neurology, University of Ulm, Ulm, Germany)
Adeline Rollin (CHU, CNR-MAJ, Labex Distalz, LiCEND Lille, France)
Gregory Kuchcinski (Univ Lille, FranceInserm 1172, Lille, France; CHU, CNR-MAJ, Labex Distalz, LiCEND Lille, France)
Maxime Bertoux (Inserm 1172, Lille, FranceCHU, CNR-MAJ, Labex Distalz, LiCEND Lille, France)
Thibaud Lebouvier (Univ Lille, France; Inserm 1172, Lille, France; CHU, CNR-MAJ, Labex Distalz, LiCEND Lille, France)
Vincent Deramecourt (Univ Lille, France; Inserm 1172, Lille, France; CHU, CNR-MAJ, Labex Distalz, LiCEND Lille, France)
Beatriz Santiago (Neurology Department, Centro Hospitalar e Universitario de Coimbra, Coimbra, Portugal)
Diana Duro (Faculty of Medicine, University of Coimbra, Coimbra, Portugal)
Maria João Leitão (Centre of Neurosciences and Cell Biology, Universidade de Coimbra, Coimbra, Portugal)
Maria Rosario Almeida (Faculty of Medicine, University of Coimbra, Coimbra, Portugal)
Miguel Tábuas-Pereira (Neurology Department, Centro Hospitalar e Universitario de Coimbra, Coimbra, Portugal)
Sónia Afonso (Instituto Ciencias Nucleares Aplicadas a Saude, Universidade de Coimbra, Coimbra, Portugal)
Annerose Engel (Clinic for Cognitive Neurology, University Hospital Leipzig, Leipzig, Germany)
Maryna Polyakova (Department for Neurology, Max Planck Institute for Human Cognitive and Brain Sciences and Clinic for Cognitive Neurology, University Hospital Leipzig, Leipzig, Germany)

15525279, 0, Downloaded from https://alz-journals.onlinelibrary.wiley.com/doi/10.1002/alz.12824 by Test, Wiley Online Library on [14/03/2023]. See the Terms and Conditions (https://onlinelibrary.wiley.com/terms-and-conditions) on Wiley Online Library for rules of use; OA articles are governed by the applicable Creative Commons License
